# Supplementary material for: Mechanical forces drive a reorientation cascade leading to biofilm self-patterning
Source: Nat Commun. 2021 Nov 17;12:6632. doi: 10.1038/s41467-021-26869-6 (PMC8599862; doi:10.1038/s41467-021-26869-6)
Supplement: Supplementary file 1 — Supplementary Information [file 41467_2021_26869_MOESM1_ESM.pdf]

Supplementary Information for

Mechanical forces drive a reorientation cascade leading to biofilm self-patterning

Japinder Nijjer, Changhao Li, Qiuting Zhang, Haoran Lu, Sulin Zhang*, Jing Yan*

*Corresponding authors. Email: jing.yan@yale.edu or suz10@psu.edu

I. Cell segmentation algorithm

After the deconvolved 3D image (Supplementary Fig. 2a, b) was binarized (Supplementary Fig. 2c), a number of different connected components (CCs), each containing multiple cells, were identified. The key difficulty in segmentation lies in the inferior resolution in the *z*-direction in confocal images as well as imperfect rejection of out-of-focus light, which makes the use of a single global threshold inadequate. Therefore, we developed an adaptive local thresholding algorithm for single-cell biofilm image analysis. Specifically, for each CC, we gradually increased the intensity threshold locally and therefore delete voxels whose intensities are below the threshold. After each local binarization step, we checked whether new CCs were generated. For each newly generated CC, if its volume was below a defined volume threshold, we extracted this CC from the image and considered this CC as the core of one individual cell. Information on this individual cell was stored and no further action was performed on this cell until the image-restoration step. If the volume of the CC was still above the volume threshold, we locally increased the threshold until the volume fell below the threshold. After the adaptive local thresholding algorithm, we obtained the cores of all individual cells; however, these cores were smaller than the original cells; they did not represent accurately the location, orientation, and size of the original cells. Therefore, we added a volume restoration step in which the deleted voxels in the segmentation step were merged with the nearest core (Supplementary Fig. 2d, e). By performing the virtual shrinking-expansion step, we maintained the accuracy in both segmentation and in measuring the shape of each bacterium.

II. Details of the agent-based simulations (ABSs)

Single cell model

We model the space occupied by a single cell and its surrounding extracellular matrix as a spherocylinder with length L, radius R and therefore volume $V = \frac{4}{3}\pi R^3 + \pi R^2 L$. We assume the cell only grows in length over time while the radius remains the same, where the volume growth obeys the exponential law $\frac{\mathrm{d}V}{\mathrm{d}t} = \gamma V$, with growth rate γ. We introduce noise during cell growth and division, by assigning different cells with slightly different growth rates, taken from a normal distribution $\gamma \sim \mathrm{N}(\gamma_0, 0.2\gamma_0)$. In the ABSs, cell growth is implemented by increasing the length L by the increment $\Delta L = \gamma \left(\frac{4}{3}R + L\right)\Delta t$, where Δt is the timestep.

Cell division is modeled as the instantaneous replacement of a mother cell when it reaches length $L_{\max}$ by two equal sized daughter cells. The slight volume loss after division is ignored to avoid contact overlapping which may cause unphysical reorientations. It follows that the initial length of a daughter cell is $L_0 = \frac{L_{\max}}{2} - R$. Given the growth law, the doubling time can be calculated as $t_{\text{double}} = \frac{1}{\gamma}\log\left(\frac{10R+6L_0}{4R+3L_0}\right)$.

Cell-cell repulsion

We only consider the repulsive, elastic contact forces between cells, mediated by the soft exopolysaccharides. Linear elastic Hertzian contact theory is applied to quantify the repulsive contact force on cell i by cell j, written as

$$\mathbf{F}_{\text{cell-cell,ij}} = -\frac{5}{2}E_0 R^{1/2}\delta_{ij}^{3/2}\hat{\mathbf{e}}_{ij} \quad \textbf{(1)}$$

where E_0 is the effective cell stiffness, δ_{ij} is the overlapping distance, and $\hat{\mathbf{e}}_{ij}$ is the unit vector parallel to the distance vector $\mathbf{d}$. The distance vector is given by connecting two contact points characterizing the smallest distance between two cell centerlines, as shown in Supplementary Fig. 6. The overlapping distance δ_{ij} is then calculated by

$$\delta_{ij} = 2R - |\mathbf{d}|, \quad \textbf{(2)}$$

and contact only occurs if $\delta_{ij} > 0$. Taking the center of mass as the reference point, the moment of contact force is

$$\mathbf{M}_{\text{cell-cell},ij} = -\frac{5}{2}s_r\hat{\mathbf{n}}_i \times E_0 R^{1/2}\delta_{ij}^{3/2}\hat{\mathbf{e}}_{ij} \quad \textbf{(3)}$$

where $\hat{\mathbf{n}}_i$ is the cell director and s_r is the parametric coordinate, along the center-line of the cell, of the contact point.

Cell-to-substrate interactions

The experimental substrate is modeled as an infinite, two-dimensional rigid plane located at $z = 0$. We again assume linear elastic Hertzian contact mechanics to quantify the repulsive contact between cells and the substrate. We also add an attractive force which depends on the contact area to account for the matrix protein mediated cell-to-surface adhesion.

To formulate the repulsive contact between the cells and the substrate, we apply a generalized Hertzian contact formula that smoothly accounts for the cell orientation dependent contact energy. In this case, the elastic deformation energy is given by $E_{\text{el},i} = E_0 R^{1/2}\delta_i^{5/2}$ and the equivalent penetration depth is given by

$$\delta_i^{5/2} = \int_{-L/2}^{L/2}\left[R^{-1/2}\left(1 - |n_{\perp,i}|^2\right)\delta^2(s) + \frac{4}{3}|n_{\perp,i}|^2\delta^{3/2}(s)\right]\mathrm{d}s \quad \textbf{(4)}$$

where $n_{\perp,i}$ is the projection of the ith cell director onto the z-axis. The overlap function $\delta(s)$ denotes the overlapping distance between the cell and the substrate at the local cell-body coordinate $-L/2 \le s \le L/2$. Then, the net force $\mathbf{F}_{\text{el},i}$ and moment $\mathbf{M}_{\text{el},i}$ from the cell-substrate elastic repulsion can be given by

$$\mathbf{F}_{\mathrm{el},i} = 2E_0 R^{1/2} \int_{L/2}^{-L/2} \hat{\mathbf{z}} \left[R^{-1/2} \left(1 - \left|n_{\perp,i}\right|^2\right) \delta(s) + \left|n_{\perp,i}\right|^2 \delta^{1/2}(s) \right] \mathrm{d}s \tag{5}$$

$$\mathbf{M}_{\mathrm{el},i} = 2E_0 R^{1/2} \int_{L/2}^{-L/2} [s\hat{\mathbf{n}}_i \times \hat{\mathbf{z}}] \left[R^{-1/2} \left(1 - \left|n_{\perp,i}\right|^2\right) \delta(s) + \left|n_{\perp,i}\right|^2 \delta^{1/2}(s) \right] \mathrm{d}s \tag{6}$$

where $\hat{\mathbf{z}}$ is the unit vector perpendicular to the substrate.

We take the adhesion energy between the cells and the substrate to be of the form $E_{\mathrm{ad},i} = -\Sigma_0 A_i$, where Σ_0 is the adhesion energy density and A_i is the equivalent contact area. The equivalent contact area is given by

$$A_i = \int_{-L/2}^{L/2} a(s)\mathrm{d}s = \int_{-L/2}^{L/2} \left[R^{1/2} \left(1 - \left|n_{\perp,i}\right|^2\right) \delta^{1/2}(s) + \pi R \left|n_{\perp,i}\right|^2 \mathrm{H}\big(\delta(s)\big) \right] \mathrm{d}s \tag{7}$$

where $\mathrm{H}(\cdot)$ is the Heaviside step function. The net adhesive force $\mathbf{F}_{\mathrm{ad},i}$ and moment $\mathbf{M}_{\mathrm{ad},i}$ are:

$$\mathbf{F}_{\mathrm{ad},i} = -\Sigma_0 \int_{-L/2}^{L/2} \hat{\mathbf{z}} \left[\frac{1}{2} R^{1/2} \left(1 - \left|n_{\perp,i}\right|^2\right) \delta^{-1/2}(s) \right] \mathrm{d}s - \hat{\mathbf{z}} \Sigma_0 \pi R \left|n_{\perp,i}\right|^2 \tag{8}$$

$$\begin{aligned} \mathbf{M}_{\mathrm{ad},i} = -\Sigma_0 \int_{-L/2}^{L/2} [s\hat{\mathbf{n}}_i \times \hat{\mathbf{z}}] \left[\frac{1}{2} R^{1/2} \left(1 - \left|n_{\perp,i}\right|^2\right) \delta^{-1/2}(s) \right] \mathrm{d}s \\ - [s_0 \hat{\mathbf{n}} \times \hat{\mathbf{z}}] \Sigma_0 \pi R \left|n_{\perp,i}\right|^2 \end{aligned} \tag{9}$$

where s_0 denotes the cell-body coordinate such that $\delta(s_0) = 0$.

Viscosity

We consider two sources of viscosity: a bulk viscous force due to the extracellular matrix environment and a surface viscous force due to the substrate. The environmental viscous force and moment are given by Stoke's law,

$$\mathbf{F}_{\mathrm{stokes},i} = -\eta_0 \mathbf{u}_i \tag{10}$$

$$\mathbf{M}_{\mathrm{stokes,i}} = -\eta_0 \int_{L/2}^{L/2} s\hat{\mathbf{n}}_i \times (\boldsymbol{\omega}_{\mathrm{i}} \times s\hat{\mathbf{n}}_i) \mathrm{d}s = -\frac{\eta_0}{12} \boldsymbol{\omega}_i L^3 \tag{11}$$

where η_0 is the environmental viscosity, $\mathbf{u}$ is the velocity of the center of mass, and $\boldsymbol{\omega}$ is the angular velocity. The substrate viscous force and moment are taken to be of the form

$$\mathbf{F}_{\mathrm{surface},i} = -\int_{-L/2}^{L/2} \frac{\eta_1 a(s)}{R} [\mathbf{u}_i(s) - (\mathbf{u}_i(s) \cdot \hat{\mathbf{z}})\hat{\mathbf{z}}] \mathrm{d}s \tag{12}$$

$$\mathbf{M}_{\mathrm{surface},i} = -\int_{-L/2}^{L/2} \frac{\eta_1 a(s)}{R} s\hat{\mathbf{n}}_i \times [\mathbf{u}_i(s) - (\mathbf{u}_i(s) \cdot \hat{\mathbf{z}})\hat{\mathbf{z}}] \mathrm{d}s \tag{13}$$

where η_1 is the viscous coefficient along the substrate.

Equations of motion

The equations of motion for each cell are given by Newton's rigid body dynamics:

$$\begin{pmatrix} \mathbf{F}_{\text{net},i} \\ \mathbf{M}_{\text{net},i} \end{pmatrix} = \begin{bmatrix} m & 0 \\ \mathbf{0} & \mathbf{I}_i \end{bmatrix} \begin{pmatrix} \dot{\mathbf{u}}_i \\ \dot{\boldsymbol{\omega}}_i \end{pmatrix} + \begin{pmatrix} \mathbf{0} \\ \boldsymbol{\omega}_i \times \mathbf{I}_i \boldsymbol{\omega}_i \end{pmatrix} \quad \textbf{(14)}$$

where $\mathbf{F}_{\text{net},i}$ and $\mathbf{M}_{\text{net},i}$ are the total force and moment vector, and $\mathbf{I}$ is the moment of inertia. All of the variables are expressed in the body-fixed coordinate system, then transformed into the global coordinate system. We add a small random noise to the net force and moment vectors of the cells at every timestep ($10^{-7}E_0R^2$ for forces and $10^{-7}E_0R^3$ for moments).

Choice of parameters for cellular dynamics

The list of physical constants, which were used to successfully capture the verticalization instability in biofilm-dwelling cells in prior work[1], are used here and given in Table S2.

Geometry of coarse-grained gel system

We model the agarose gel using a coarse-grained particle-based approach. We treat the gel as a collection of spherical particles, each with radius R_{gel}. We treat the interactions of the spheres using a spring network model to recapitulate the elastic behavior of the hydrogel. The pairwise interaction energy between the gel particles is $E_{\text{gel},2} = \Sigma_{ij} \frac{k_r}{2} (\xi_{ij} - \xi_0)^2$, where ξ_{ij} is the distance between particle i and j, ξ_0 is the equilibrium distance, and k_r is the spring constant. Furthermore, to impart a shear modulus to the system, we also include a three-body interaction energy where $E_{\text{gel},3} = \Sigma_{ijk} \frac{k_\zeta}{2} (\zeta_{ijk} - \zeta_0)^2$, where ζ_{ijk} is the bond angle formed by particle i, j, and k.

Interactions between gel particles and cells

We again apply linear elastic Hertzian contact theory to describe the repulsive interaction between the coarse-grained gel particles and the cells. Similar to the contact between cells, the elastic contact energy can be written as $E_{\text{gel-cell},ij} = E_1 \left(\frac{1}{R} + \frac{1}{R_{\text{gel}}}\right)^{-1/2} \delta_{ij}^{5/2}$, where E_1 is the contact stiffness between gel and cell, and δ_{ij} is the overlapping distance between the ith gel particle and jth cell. The contact stiffness E_1 can be given by the relation $\frac{1}{E_1} = \frac{1-\mu_{\text{gel}}^2}{Y_{\text{gel}}} + \frac{1-\mu_{\text{cell}}^2}{Y_{\text{cell}}}$, where Y and μ are Young's modulus and Poisson's ratio, respectively.

Interactions between the gel particles and the substrate

There exist two types of interactions between the coarse-grained gel particles and the substrate. First, the gel particles make elastic contact with the substrate, again described by Hertzian contact theory for the contact between a sphere and a flat surface, where the elastic contact energy is given by $E_{\text{gel-surface},i} = E_1 R_{\text{gel}}^{1/2} \delta_i^{5/2}$. Second, we introduce adhesion between the gel particles and substrate which provides an energy barrier to delamination; we take the energy to be of the form $E_{\text{ad,gel},i} = -\Sigma_1 A_{\text{gel},i}$, where Σ_1 is the adhesion energy density. The equivalent contact area is given by $A_{\text{gel},i} = \pi R_{\text{gel}} \delta_i$, where δ_i is the overlap between the gel particle and the substrate.

Equation of motion

We only consider the three translational degrees of freedom for gel particles, neglecting the rotational degrees of freedom. Therefore, the equations of motion are given by Newton's second

law $\mathbf{F}_{\text{tot},i} = m\mathbf{a}_i$, where $\mathbf{F}_{\text{tot},i}$ is the net force and $\mathbf{a}_i$ is the acceleration. To prepare the initial amorphous stress-free geometry, we begin with a body-centered cubic crystalline geometry with lattice parameter a, where $a = 1.3R_{\text{gel}}$. Subsequently, we assigned the system with an initial temperature of $300K$ and annealed it until it reached a final configuration that is amorphous and stress-free.

Choice of parameters

The spring constant k_r and equilibrium length ξ_0: The Young's modulus of the coarse-grained gel system is given by $Y = \frac{1}{V}\frac{\partial^2(E_{\text{gel},2}+E_{\text{gel},3})}{\partial\epsilon^2} \simeq \frac{k_r}{2\xi_0}2$, under the condition $k_\zeta \ll k_r$. Generally, a smaller ξ_0 leads to a denser gel system and better approximation to a continuum solid. Here we choose $\xi_0 = 0.6$ μm as a result of a trade-off between simulation quality and computational cost, as the simulation time is proportional to $\frac{1}{\xi_0{}^3}$. This ξ_0 value leads to $k_r = 6 \times 10^{-3}\text{Nm}^{-1}$, corresponding to the experimentally measured $Y = 5kPa$.

Radius of coarse-grained gel particle R_{gel}: In order to mimic the continuum constraints posed by the hydrogel in the experiment, the coarse-grained system should not have significant defects larger than the volume of a single cell, which means that R_{gel} should be larger than ξ_0. On the other hand, R_{gel} cannot be significantly larger than the cell radius R, as this will introduce unphysical contacts at the biofilm-gel interface. Taking both requirements into consideration, we choose $R_{\text{gel}} = 1.0$ μm, which is nearly double the equilibrium distance $\xi_0 = 0.6$ μm and we keep $R_{\text{gel}} \approx R$.

Cell-gel contact modulus E_1: Hertzian contact mechanics gives the equivalent contact modulus between two elastic bodies by $\frac{1}{E_1} = \frac{1-\mu_{cell}^2}{Y_{cell}} + \frac{1-\mu_{gel}^2}{Y_{gel}}$, where Y is the Young's modulus and μ is Possion's ratio of the two contacting elastic bodies. Given the bulk rheology measurement $Y_{\text{cell}} = 500$ Pa, $\mu_{\text{cell}} = \mu_{\text{gel}} = 0.49$, and $Y_{\text{gel}} = 5$ kPa[3,4], it follows $E_1 = 600$ Pa. In the simulations, we choose $E_1 = 1500$ Pa, somewhat larger than E_0, to avoid the unphysical situation in which cells penetrate into the gel during growth.

The three-body interaction parameter k_ζ and equilibrium angle ζ_0: To make the amorphous coarse-grained gel system stable, we choose $\zeta_0 = 120°$ to force the system to deviate from its original cubic configurations. We select k_ζ such that we attain incompressible solid behavior in the gel $G = Y/3$, where G is the shear modulus and Y is Young's modulus.

These parameters are listed in Table S3.

III. Single-cell surface adhesion stability analysis

Previous studies have described how cells in 2D bacterial colonies can be peeled from the substrate through a buckling-like instability[5–8]; however, the reason why biofilm-dwelling cells self-organize into a verticalized core, that is, remain attached but oriented away from the substrate, remains to be shown. In this section, we consider the stability of surface-adhered cells subject to compression from neighboring cells to determine why surface adhesion facilitates *stably* surface-anchored, verticalized cells. Specifically, we consider a minimal model examining the instability of a single spherocylindrical cell that is compressed by its neighbors, following the same physical

model described in the agent-based simulation section above; however, in our theoretical analysis we neglect any external gel confinement, and only consider the effects of bulk viscosity for analytical tractability. We assume that the central cell is not growing and instead we mimic the effects of growth-induced compression by bringing neighboring cells closer together. The central cell initially starts out parallel to the substrate in close contact with its neighbors, and the peripheral neighboring cells are brought closer to each other to squeeze the central cell (see the schematic in Figure 2f). We find that the central cell evolves through four phases upon increasing compression: **I.** the cell remains horizontal and the elastic potential energy in the cell increases; **II.** the central cell rotates from horizontal to vertical and all potential energy is consequently released; **III.** the central cell remains vertical, and the elastic potential energy increases again when the outer cells touch the center cell again; and **IV.** the central cell becomes unstable and is ejected from the substrate. We confirm this sequence with a simple 2D agent-based model where cells are confined to the x-z plane. The transition from I to II corresponds to a "verticalization" instability[1], from II to III corresponds to a trivial geometric transition, and from III to IV corresponds to a "pinch-off" instability. Note that the pinch-off instability is locally (marginally) stable, but under sufficiently large z-perturbations, the central cell gets ejected from the substrate. Experimentally, we expect that many factors could contribute to the perturbations including but not limited to: deviation of the cell shape from a perfect spherocylinder, fluctuating protein bonds, and variations in the adhesion protein concentration, etc. In what follows below, we consider these two instabilities in more detail paying specific attention to the role of the cell-to-surface adhesion.

The equations of motion for the position $\mathbf{r} = (x, y, z)$, and director $\hat{\mathbf{n}} = (n_x, n_y, n_z) = \mathbf{n}_{\parallel} + n_{\perp}\hat{\mathbf{z}}$ of a cell written using Lagrangian mechanics, are:

$$\eta_0 l \dot{\mathbf{r}} = -\frac{\partial \sum E}{\partial \mathbf{r}},$$
$$\frac{\eta_0 l^3 \dot{\hat{\mathbf{n}}}}{12} = -\frac{\partial \sum E}{\partial \hat{\mathbf{n}}} + \lambda \frac{\partial F}{\partial \hat{\mathbf{n}}}, \quad \textbf{(15A,B)}$$

where λ is a Lagrange multiplier corresponding to the constraint $F = |\hat{\mathbf{n}}| - 1 = 0$. Here $\sum E = E_{el} + E_{ad} + E_{cell-cell}$ is the total potential energy and includes cell-to-cell repulsion, as well as cell-to-surface adhesion and repulsion terms. These surface interaction terms are given by

$$E_{el} = E_0 R^{1/2} \int_{-L/2}^{L/2} \left[R^{-1/2} |\mathbf{n}_{\parallel}|^2 \delta^2(s) + \frac{4}{3} |n_{\perp}|^2 \delta^{3/2}(s) \right] \mathrm{d}s,$$
$$E_{ad} = -\Sigma_0 \int_{-L/2}^{L/2} \left[R^{1/2} |\mathbf{n}_{\parallel}|^2 \delta^{1/2}(s) + \pi R |n_{\perp}|^2 \mathrm{H}(\delta(s)) \right] \mathrm{d}s. \quad \textbf{(16A,B)}$$

In phase I, in the absence of any compression from neighboring cells, the balance of surface adhesion and repulsion leads to an equilibrium cell configuration $n_{\perp} = 0$ at an apparent height

$$z_{0,h} = R\left(1 - \left(\frac{\tilde{A}}{4}\right)^{\frac{2}{3}}\right), \quad \textbf{(17)}$$

where $\tilde{A} = \Sigma_0 / R E_0$ is the dimensionless adhesion coefficient. In the presence of compression, we consider perturbations away from this stable point.

To simplify the analysis, we assume that all neighboring cells compress and lead equally to a build-up of elastic energy or pressure in the central cell of interest ($E_{\mathrm{cc}} = E_0 R^{1/2} \delta^{5/2}$ where δ is the overlap distance between two cells). We assume hexagonal close packing around the caps of the cell and square close packing around the cylindrical portion of the cell leading to a maximum of $\sim 6 + L_{\max}/R$ neighbors compressing the central cell (see schematic in Supplementary Fig 10a). Finally, we make the simplifying assumption that the *dominant* compression that leads to verticalization is due to the two neighbors pushing on the two ends of the central cell. Note that in practice the exact configuration around the central cell will vary, leading to a stochastically varying stress field; our analysis is meant to provide an estimate of the relative energy barriers between the verticalization and pinch-off instabilities.

Combining the cell-surface adhesion, cell-surface repulsion, and cell-cell repulsion terms, we find the evolution equation for small $n_\perp$ is given by

$$\dot{n}_\perp = \Omega(\delta) n_\perp, \quad \textbf{(18)}$$

where the sign of Ω determines the stability of the fixed-point. Supplementary Figure 10c shows plots of Ω for different dimensionless adhesion coefficients. For small values of $\tilde{A}$, which are most physically relevant, the zeros of Ω are only weakly dependent on $\tilde{A}$. This analysis suggests that the verticalization instability only weakly depends on adhesion, that is, the energy barrier to verticalization is nearly constant. This counterintuitive result arises because for $\tilde{A} \ll 1$, adhesion does not impart a significant torque on the cell. Instead, while compression from peripheral cells is destabilizing, cell-surface *repulsion* acts to provide a stabilizing torque.

In phase III, again assuming no compression from neighboring cells, the balance of surface adhesion and repulsion leads to an equilibrium verticalized configuration $n_\perp = 1$ with height

$$z_{0,v} - \frac{L}{2} = R\left(1 - \left(\frac{3\pi\tilde{A}}{4}\right)^{2/3}\right). \quad \textbf{(19)}$$

Note that the effective penetration depth (the degree to which the soft cell is flattened against the substrate) is larger for verticalized cells than for horizontal cells. This difference in penetration depth occurs due to the difference in geometry between a hemispherical cap and a cylindrical body and how the cell-surface adhesion and cell-surface repulsion energies scale with the penetration depths.

Again, we consider compression due to neighboring cells (at a height $z_{0,h}$). In this case, the footprint of the verticalized cell is a circle and so we assume 6 neighboring cells that are hexagonally closed packed around it (Supplementary Fig 10b). In this configuration all neighbors contribute equally to the pinch-off of the central cell. Here we look for finite-sized perturbations away from the fixed point; the evolution equation of the height of the cell of interest is given by

$$\dot{z} = w(\delta, z). \quad \textbf{(20)}$$

We look for points (δ, z) such that $w(\delta, z) > 0$. We only consider points $0 \leq \frac{L}{2} + R - z < R - z_{0,h}$ as points $z - \frac{L}{2} < z_{0,h}$ results in no net vertical force such that the cell of interest remains

stably attached to the substrate. Specifically, we focus on points very close to $z = L/2 + R$ to determine the critical energy required to produce marginally unstable cells. Supplementary Figure 10d shows plots of $w(\delta, L/2 + 0.999R)$ for different adhesion coefficients. In contrast to the verticalization transition, in this case we find a strong dependence of the zeros of w on $\tilde{A}$; therefore, the energy barrier to pinch-off depends strongly on cell-to-surface adhesion.

We combine these two stability analyses into a single plot of the energy landscape experienced by the central cell for different adhesion energies (Supplementary Figure 10e). We see that for very small adhesion energies, pinch-off is energetically favored over verticalization, whereas for larger adhesion energies, pinch-off is energetically more costly than verticalization. Importantly, we find that for the parameters listed in Table S2, the critical energy corresponding to the verticalization instability is much smaller than that of the pinch-off instability, leading to preferential verticalization of horizontal cells rather than pinch-off of verticalized cells in a population of cells in the basal layer (Fig. 2f). This analysis explains the observed positive correlation between the adhesion energy and the fraction of stably verticalized cells (Supplementary Fig. 5, 7, 8): adhesion energy affects the stability of cells with respect to the pinch-off instability but much less so to the verticalization instability.

IV. Continuum model for growth-induced macroscopic cell ordering

In this section, we present a minimal coarse-grained description to explain the growth and self-organization observed in the basal layer of *V. cholerae* biofilms. We first consider the simpler case with one population of cells that either generate in-plane growth or not, before considering the complete two-phase model which accounts for pressure-dependent cell verticalization and out-of-plane growth.

<u>*Theoretical framework*</u>

We limit our consideration to the basal plane of the biofilm and assume it is a growing, quasi-2D system with *mesoscopic* nematic order tensor $\mathbf{Q} = 2\langle \hat{\mathbf{n}} \otimes \hat{\mathbf{n}} - \mathbf{I}/2 \rangle$[9]. Based on its symmetries, in 2D, $\mathbf{Q}$ can also be written as

$$\mathbf{Q} = q \begin{bmatrix} \cos(2\theta) & \sin(2\theta) \\ \sin(2\theta) & -\cos(2\theta) \end{bmatrix} \quad \textbf{(21)}$$

where θ is the angle of the average head-less director and q is the scalar order parameter, which quantifies the degree to which the cells are locally ordered. In the analysis below we will assume that this scalar order parameter is constant.

We assume the bacterial cells grow with a uniform growth rate γ but that the growth can be out-of-plane, resulting in a spatially varying in-plane growth rate $g(\mathbf{r})$. Taking the cell density as c, and the coarse-grained growth-induced velocity field as $\mathbf{u}$, mass conservation requires

$$\partial_t c + \nabla \cdot (c\mathbf{u}) = gc. \quad \textbf{(22)}$$

The density is nearly uniform in the experiment, that is, $c(\mathbf{r}) = c_0 + \epsilon c_1(\mathbf{r})$, where $\epsilon \ll 1$. To leading order, Eq. **(22)** becomes

$$\nabla \cdot \mathbf{u} = g. \quad \textbf{(23)}$$

The growth-induced velocity field is found by integrating the spatially dependent growth rate. In this analysis, we will neglect density fluctuations c_1, but it can be related to the fluctuation in local pressure due to varying local configurations.

The evolution of the nematic order tensor is described by the Beris-Edward equation[10]

$$(\partial_t + \mathbf{u} \cdot \nabla)\mathbf{Q} = \lambda\mathbf{E} - (\boldsymbol{\omega} \cdot \mathbf{Q} - \mathbf{Q} \cdot \boldsymbol{\omega}) + \Gamma\mathbf{H}, \tag{24}$$

where λ is the flow-alignment parameter and $\lambda > 0$ for rod-shaped objects that tend to align in shear. Here $\mathbf{E} = \frac{1}{2}(\nabla\mathbf{u} + \nabla\mathbf{u}^T - (\nabla \cdot \mathbf{u})\mathbf{I})$ and $\boldsymbol{\omega} = \frac{1}{2}(\nabla\mathbf{u} - \nabla\mathbf{u}^{\mathrm{T}})$ are the traceless strain-rate and vorticity tensors and $\mathbf{H}$ is the molecular field. $\Gamma\mathbf{H}$ relaxes $\mathbf{Q}$ towards a bulk state with minimal spatial variation in the director. However, we do not expect $\Gamma\mathbf{H}$ to play an important role in biofilm ordering because biofilm-dwelling cells secrete exopolysaccharides which are soft and deformable and act as a "cushion" between cells. This can be seen in the biofilm from the ΔBC mutant (Fig. 1e) in which minimal local alignment was observed. Contrast this with the $\Delta vpsL$ mutant and other bacterial colonies[11–13] where there is significant local alignment (Fig. 1f) when no exopolysaccharides are present. Finally, force balance requires

$$\nabla \cdot \boldsymbol{\Pi} = \eta\mathbf{u}, \tag{25}$$

where $\boldsymbol{\Pi}$ is the stress tensor in the biofilm, whose divergence is balanced by surface friction. Here we have assumed that energy dissipation is dominated by surface friction rather than viscous dissipation inside the biofilm, corresponding to the so-called "dry" limit[14]. We take the frictional force density to be linearly proportional to the velocity and the friction coefficient η to be isotropic and constant. In many active nematic systems, the active stress is anisotropic and dependent on $\mathbf{Q}$. For instance, in motile cell colonies, cells generate anisotropic active stresses[15–17] while in growing bacterial colonies a number of different constitutive relations have been proposed[11,12,18,19]. Although at the microscopic scale, these forces may be anisotropic, on the mesoscopic scale pertaining to our theory, they are expected to be isotropic on average. This leads to the assumption of an isotropic stress field which is balanced by surface friction,

$$\nabla p = \eta\mathbf{u}. \tag{26}$$

Pressure in this system arises due to compression from neighboring cells, mediated by the exopolysaccharides, which arises from cell proliferation.

To close the model, we impose the following set of boundary and initial conditions. By symmetry, we expect that the velocity goes to zero at the center of the biofilm,

$$\mathbf{u}(\mathbf{0}, t) = \mathbf{0}, \tag{27}$$

that is, the center of the biofilm does not move and

$$\frac{\partial\mathbf{Q}}{\partial r}(\mathbf{0}, t) = \mathbf{0}. \tag{28}$$

We also assume that the pressure accumulates towards the center of the biofilm and that at the outer edge of the biofilm the pressure is zero,

$$p(r = r_e, t) = 0, \tag{29}$$

where r_e denotes the edge of the biofilm. The outer radius evolves through the kinematic condition

$$\frac{dr_e}{dt} = u_r(r_e). \quad \textbf{(30)}$$

Finally, we assume that the orientation of the biofilm is described by some initial order parameter tensor

$$\mathbf{Q}(r, \psi, t = 0) = \mathbf{Q_0}(r, \psi), \quad \textbf{(31)}$$

which can also be written as $\theta(r, \psi, t = 0) = \theta_0(r, \psi)$.

Equations **(23)**, (**24)**, (**26)** and boundary conditions and initial conditions Eq. (**27)-(31)** form the basis of the theoretical model. In the following subsections, we consider a few idealized cases to determine under which conditions biofilms self-organize into an aster pattern. Throughout, we will also make the simplifying assumption that all variables except for $\boldsymbol{Q}$ are axisymmetric and that there is no azimuthal flow, $\mathbf{u} = u_r \hat{r}$.

<u>*Uniform growth limit*</u>

If all growth is in-plane, then $g = \gamma$ is uniform and constant. Integrating Eq. (**23)** we find the velocity field is

$$u_r = \frac{\gamma r}{2}, \quad \textbf{(32)}$$

and the strain-rate and vorticity tensors are exactly zero, $\mathbf{E} = \boldsymbol{\omega} = \mathbf{0}$. Solving Eq. (**24)** yields

$$\mathbf{Q}(r, \psi, t) = \mathbf{Q}_0\left(re^{-\frac{\gamma t}{2}}, \psi\right). \quad \textbf{(33)}$$

In this case, **Q** *is simply stretched by growth, and there is no tendency for* **Q** *to rotate or align in any given direction*. An initially isotropic system will remain isotropic as it grows (Supplementary Fig. 14a-c)[12,20]. This is what happens in the 2D expansion of bacterial colonies, which remain macroscopically isotropic as they grow. Although microscopic forces can align cells locally, they cannot introduce any long-range order. Note that in two dimensions axisymmetric growth is a special case where there is no net strain in the system: if instead the system were confined into a rectangular channel, where growth is unidirectional, the strain rate tensor would not be zero and therefore there would be net alignment along the direction of the channel[18,19,21,22].

<u>*Alignment due to a growth void*</u>

Now suppose there is a radius r_0 inside which the cells have verticalized and are angled out of plane. These cells do not contribute to the growth of the basal plane and so

$$g(r) = \begin{cases} 0 \text{ if } r < r_0 \\ \gamma \text{ if } r \geq r_0 \end{cases}. \quad \textbf{(34)}$$

Substituting into Eq. (**23)**, the growth-induced velocity is

$$u_r = \begin{cases} 0 & \text{if } r < r_0 \\ \gamma(r - r_0^2/r)/2 & \text{if } r \geq r_0 \end{cases}. \quad \textbf{(35)}$$

The corresponding strain rate and vorticity tensors in polar coordinates ($\mathbf{E}_p, \boldsymbol{\omega}_p$) are

$$\mathbf{E}_p = \mathbf{0}, \qquad \boldsymbol{\omega}_p = \mathbf{0} \text{ if } r < r_0 \quad \textbf{(36)}$$

$$\mathbf{E}_p = \begin{bmatrix} \left(\frac{\partial u_r}{\partial r}\right) - \frac{\gamma}{2} & 0 \\ 0 & \frac{u_r}{r} - \frac{\gamma}{2} \end{bmatrix}, \boldsymbol{\omega}_\mathbf{p} = \mathbf{0} \text{ if } r \geq r_0.$$

Under uniform growth $\partial u_r/\partial r$ and u_r/r are equal to $\gamma/2$, leading to no net deviatoric strain-rate. In contrast, in the presence of a growth void, these two terms become unequal, leading to a net deviatoric strain-rate. This is because the radial gradient in velocity is no longer balanced by azimuthal stretching due to outward expansion. For a velocity of the form given in Eq. (**35**), the strain rate and vorticity tensors in polar coordinates are

$$\mathbf{E}_p = \mathbf{0}, \qquad \boldsymbol{\omega} = \mathbf{0} \text{ if } r < r_0$$
$$\mathbf{E}_p = \frac{\gamma r_0^2}{2r^2}\begin{bmatrix} 1 & 0 \\ 0 & -1 \end{bmatrix}, \qquad \boldsymbol{\omega} = \mathbf{0} \text{ if } r \geq r_0. \tag{37}$$

Next, the nematic order parameter $\mathbf{Q}$ can be transformed to a polar coordinate system, given by

$$\mathbf{Q}_p = \mathbf{R}^\mathrm{T}\mathbf{Q}\mathbf{R} = q\begin{bmatrix} \cos(2(\theta-\psi)) & \sin(2(\theta-\psi)) \\ \sin(2(\theta-\psi)) & -\cos(2(\theta-\psi)) \end{bmatrix}, \tag{38}$$

where ψ is the azimuthal angle and $\mathbf{R} = \begin{bmatrix} \cos\psi & -\sin\psi \\ \sin\psi & \cos\psi \end{bmatrix}$ is the coordinate transformation matrix from cartesian to polar coordinates.

Finally, given $\mathbf{E}_p$, $\boldsymbol{\omega}_p$ and $\mathbf{Q}_p$, the matrix form of Eq. **(24)** in polar coordinates is

$$q\left(\partial_t + u_r\frac{\partial}{\partial r}\right)\begin{bmatrix} \cos(2(\theta-\psi)) & \sin(2(\theta-\psi)) \\ \sin(2(\theta-\psi)) & -\cos(2(\theta-\psi)) \end{bmatrix} = \frac{\lambda\gamma r_0^2}{2r^2}\begin{bmatrix} 1 & 0 \\ 0 & -1 \end{bmatrix}, \tag{39}$$

for $r \geq r_0$, which yields two scalar equations

$$-\sin(2(\theta-\psi))\left(\frac{\partial\theta}{\partial t} + u_r\frac{\partial\theta}{\partial r}\right) = \frac{\lambda\gamma r_0^2}{4qr^2},$$
$$\cos(2(\theta-\psi))\left(\frac{\partial\theta}{\partial t} + u_r\frac{\partial\theta}{\partial r}\right) = 0. \tag{40A,B}$$

Defining $\Theta = (\theta - \psi)$ and combining the two scalar equations above, we find that for $r \geq r_0$,

$$\partial_t\Theta + u_r\partial_r\Theta = -\frac{\lambda\gamma r_0^2}{4qr^2}\sin(2\Theta). \tag{41}$$

By first ignoring the advective term, we see that $\partial_t\Theta \sim -\sin(2\Theta)$ which has stable fixed points at $\Theta = n\pi$ or $\theta = \psi + n\pi$, characteristic of an aster pattern. *In other words, the rightmost term, which arises due to gradients in the growth-induced velocity field, drives cells to reorient towards an aster pattern.* The same observation was made during inward growth in bacterial colonies where deviation of the velocity field from that of isotropic expansion resulted in radial alignment of cells[20].

More generally, if we suppose that instead of a growth void, there is a general differential growth rate: γ for $r < r_0(t)$ and $\gamma + \Delta\gamma$ for $r \geq r_0(t)$ then the evolution equation for Θ for $r > r_0$ is given by

$$\partial_t \Theta + u_r \partial_r \Theta = -\frac{\lambda \Delta\gamma r_0^2}{4qr^2} \sin(2\Theta). \tag{42}$$

If the biofilm has a faster growing outer rim $\Delta\gamma > 0$, the bacteria will tend to organize into an aster pattern. If, on the other hand, the biofilm has a faster growing core, i.e. $\Delta\gamma < 0$, Θ will have stable fixed points at $\Theta = n\pi + \pi/2$, characteristic of a vortex pattern. This means that biofilms that exhibit excess growth in the core will be driven to form a vortex.

Returning to Eq. **(41)**, we solve it by the method of characteristics, assuming some initial maximum biofilm radius r_e, which yields

$$\cot[\Theta(r,\psi)] = \cot[\Theta_0(r',\psi)] \exp\left[\frac{\lambda}{2q}\left(\gamma t + \log\left(\frac{r'^2}{r^2}\right)\right)\right], \tag{43}$$

where $r' = \left((r^2 - r_0^2)e^{-\gamma t} + r_0^2\right)^{1/2}$ and $\Theta_0 = \theta_0 - \psi$ corresponds to the angular representation $\mathbf{Q}_0$. Plots of Eq. **(43)** are given in Supplementary Figures 14d-f. Note that, although cells tend to align in an aster pattern, the aligning torque becomes weaker as cells are advected outwards. Therefore, S does not necessarily reach a value of 1 (Supplementary Fig. 14g). Depending on the initial size of the growth void as well as the flow alignment parameter, different degrees of radial alignment are reached in the final state, with a larger r_0/r_e leading to overall more radial alignment.

To be more general, now instead of a finite void, suppose there is an arbitrary velocity field $u_r(r,t)$ corresponding to an arbitrary growth rate $g(r,t)$. Following the same procedure as above, we find the evolution equation for Θ is

$$\partial_t \Theta + u_r \partial_r \Theta = -f(r,t)\sin(2\Theta), \tag{44}$$

where $f = (\lambda r/4q)\partial_r(u_r/r)$ quantifies the aligning torque due to gradients in the flow field. Equation **(44)** thus describes a generalized time-evolution equation for the orientation field due to gradients in the flow velocity.

Two-phase active nematic model for cell verticalization and alignment

We expand the active nematic model in the previous subsection to include the verticalization dynamics in a two-phase model consisting of vertical and horizontal cells. Consider a quasi-2D layer of cells on a substrate where the cells are either parallel (horizontal) or tilted (vertical) with respect to the substrate and let ρ denote the fraction ($0 \le \rho \le 1$) of vertical cells and $1-\rho$ therefore denote the fraction of horizontal cells. Horizontal cells proliferate and generate their progeny in the basal layer of interest, while vertical cells do not contribute to the expansion to the basal layer. The growth-induced stress generated by the horizontal cells also cause them to verticalize with a pressure dependent rate $C(p)$. The evolution equation for the fraction of these two populations of cells is therefore given by:

$$\begin{gathered}\partial_t \rho + \nabla\cdot(\mathbf{u}\rho) = C(p)(1-\rho)\\ \partial_t(1-\rho) + \nabla\cdot\big(\mathbf{u}(1-\rho)\big) = \gamma(1-\rho) - C(p)(1-\rho).\end{gathered} \tag{45A,B}$$

Given that cell verticalization requires a minimum threshold pressure p_t, we make the simple assumption that the conversion rate is a linear function of the excess pressure

$$C(p) = \beta\frac{p-p_t}{p_t} H(p-p_t), \tag{46}$$

where H denotes the Heaviside function. Combining Eq. (**45A,B)**A and (**45A,B)**B yields

$$\nabla \cdot \mathbf{u} = \gamma(1-\rho). \quad \textbf{(47)}$$

We initiate the biofilm with some initial radius $r_{e,0}$ and assume all cells are horizontal to begin with, $\rho(r, t=0) = 0$. Finally, we assume symmetry at the origin which requires $\frac{\partial \rho}{\partial r} = 0$ and that ρ is axisymmetric. Equations (**26**), (**44)-(47)** with the above mentioned initial and boundary conditions make up the full two-phase continuum model for cellular ordering driven by verticalization.

Non-dimensionalization

We non-dimensionalize Eq. (**26**), (**44)(47)** by the following dimensional scales: time $t_d = 1/\gamma$, pressure $p_d = p_t$, length $r_d = (p_t/\eta\gamma)^{1/2}$ and velocity $u_{r,d} = r_d\gamma$, which yields

$$\frac{\partial \tilde{p}}{\partial \tilde{r}} = \tilde{u}_r, \quad \textbf{(48)}$$

$$\frac{\partial \Theta}{\partial \tilde{t}} + u_{\tilde{r}}\frac{\partial \Theta}{\partial \tilde{r}} = -\tilde{L}\tilde{r}\frac{\partial \tilde{u}_r/\tilde{r}}{\partial \tilde{r}}\sin(2\Theta), \quad \textbf{(49)}$$

$$\frac{\partial \rho}{\partial \tilde{t}} + \frac{1}{r}\frac{\partial(\tilde{r}\tilde{u}_r\rho)}{\partial \tilde{r}} = \tilde{\beta}(1-\rho)(p-1)H(p-1), \quad \textbf{(50)}$$

$$\frac{1}{r}\frac{\partial(\tilde{r}\tilde{u}_r)}{\partial \tilde{r}} = 1-\rho. \quad \textbf{(51)}$$

with boundary conditions $p(\tilde{r}_e) = 0$, $\tilde{u}_r(0) = 0$, $d_t\tilde{r}_e = \tilde{u}_r(\tilde{r}_e)$, $\partial_{\tilde{r}}\Theta|_{\tilde{r}=0} = 0$, and $\partial_{\tilde{r}}\rho|_{\tilde{r}=0} = 0$ and initial conditions $\Theta(\tilde{r}, \psi, 0) = \Theta_0(\tilde{r}, \psi)$ and $\rho(\tilde{r}, 0) = 0$. At the boundary, when solving for Θ, we impose a sharp velocity gradient $\Delta\tilde{u}_r/\Delta\tilde{r} \approx -\tilde{u}_r(\tilde{r}_e)/(\tilde{l}_{\text{Cell}}/2)$ where $\tilde{l}_{\text{Cell}}$ is the average length of a cell, to account for the fact that across the boundary cells there is a sharp velocity gradient which tends to align cells tangentially to the boundary[7,12]. There are two key parameters that control the evolution of the system: the dimensionless flow alignment parameter $\tilde{L} = \lambda/4q$ and the dimensionless verticalization rate $\tilde{\beta} = \beta/\gamma$. The flow alignment parameter dictates how quickly the cells will align to a straining flow, while the verticalization rate dictates how quickly horizontal cells are converted to vertical cells. For simplicity, we assume that the only distinguishing feature of the nonadherent mutant is that $\beta = 0$; however, we would also expect that in the absence of adhesion, η would also be smaller and lead to an underestimation of the characteristic length-scale.

Choice of parameters

We choose the following set of parameters when solving for the evolution of the system.

Growth rate γ: The number of bacteria in a three-dimensional biofilm was measured over time and the growth rate was found to be $\gamma \approx 0.6$ hr.$^{-1}$

Threshold verticalization pressure p_t: From the theoretical verticalization analysis, using experimentally calibrated parameters, it was found that the critical cell overlap distance at which point verticalization happens is $\delta \sim 0.125$ µm. From this value, the critical cell-cell contact pressure can be calculated as $p_d = 4E\delta^{\frac{1}{2}}/(3\pi R^{*1/2})$ where E is the contact modulus of the cells which we estimate to be 500 Pa using bulk rheological measurements (shear modulus of a bulk WT* biofilm was measured to be ~200 Pa) and $R^* = R/2 = 0.4$ µm. This yields a threshold pressure of $p_t \sim 130$ Pa.

Friction coefficient η: Using the surface viscosity η_1 defined in Table S2, we estimate the friction coefficient as $\eta = \eta_1/A$ where A is the surface footprint of the cell which we estimate to be $A \sim 3$ µm^2. The friction coefficient is therefore $\eta \sim 7 \times 10^4$ Pa s/µm^2.

Characteristic length scale r_d: The characteristic length-scale $r_d = (p_t/\eta\gamma)^{1/2}$ in the system corresponds to the characteristic size of biofilm when verticalization begins. For the parameters defined above, this length scale is $r_d \sim 3.4$ µm.

Mean cell length l_{Cell}: Taking the average of the largest and smallest cylindrical sections of the spherocylindrical cells (corresponding to right before and right after division) gives a length of $l_{\text{Cell}} = 1.8$ µm.

Initial radius of biofilm $r_{e,0}$: We initialize the biofilm to have an area equivalent to the footprint of a single cell. This gives a value $r_{e,0} \sim 1$ µm.

We solve the dimensionless Eq. (**48**)-(**51**) using a finite difference scheme with explicit time-stepping, and up-winding of advective terms. Example solutions are given in Supplementary Figures 19 and 20. We fit the two unknown dimensionless parameters to the experimental results and find that $\tilde{\beta} \approx 2.5$ and $\tilde{L} \approx 1.5$. These parameters compare favorably to what has been measured before. Namely, Beroz *et al.*[1] measured in ABS that in a biofilm growing without a confining agarose gel, $\beta \sim 2.5$ hr.$^{-1}$. Here we find a somewhat smaller value, $\beta \sim 1.5$ hr.$^{-1}$, likely owing to the fact that confinement due to the agarose gel tends to impart normal stresses that suppress verticalization. Nonetheless, we find that the results are only weakly dependent on β for $\beta > \gamma$ (Fig. 4a-d; Supplementary Fig. 19d-k). Finally, we estimate q by measuring the cell-cell orientation correlation for neighboring cells in the basal layer of the experimental biofilms and find $q \sim 0.5$ and so $\lambda \sim 3$. Measuring the intrinsic flow alignment parameter is difficult as it is dependent on many factors including the size, shape, aspect ratio and local nematic order[23], however, a value of 3 is in reasonable agreement with values of 0.3-2 reported for other growing and non-growing bacterial systems[11,18,24].

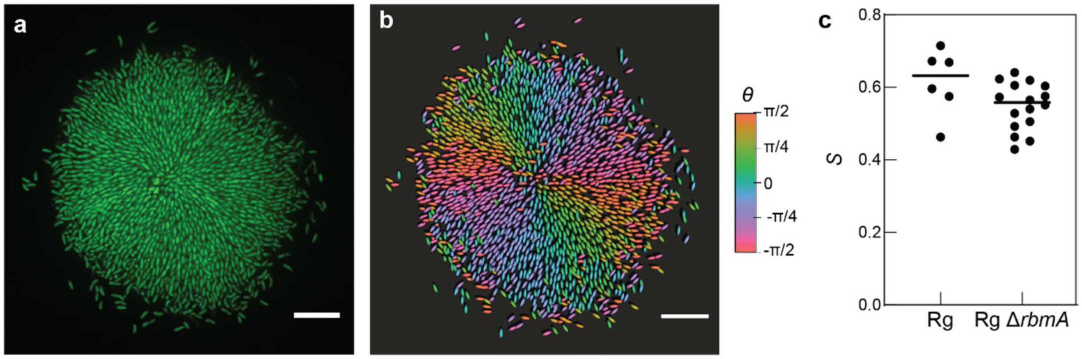

Supplementary Fig. 1| Self-patterning in biofilms formed by rugose (Rg) wild-type *V. cholerae* biofilms. **a**, Cross-sectional view of the basal plane of a rugose biofilm with an intact *rbmA* gene. **b**, The same biofilm reconstructed where cells are colored based on the angle of the in-plane director. Scale bars, 10 μm. **c**, Radial order parameter S in biofilms formed by the rugose wild-type and RgΔ*rbmA* strain (labeled as WT* in the main text). Lines correspond to median values. We find no statistical difference between the two strains ($P = 0.1414$; unpaired two-sided *t*-test with Welch's correction) under this growth condition, suggesting that the presence of the cell-to-cell adhesion protein does not interfere with the global organization mechanism. Source data are provided as a Source Data file.

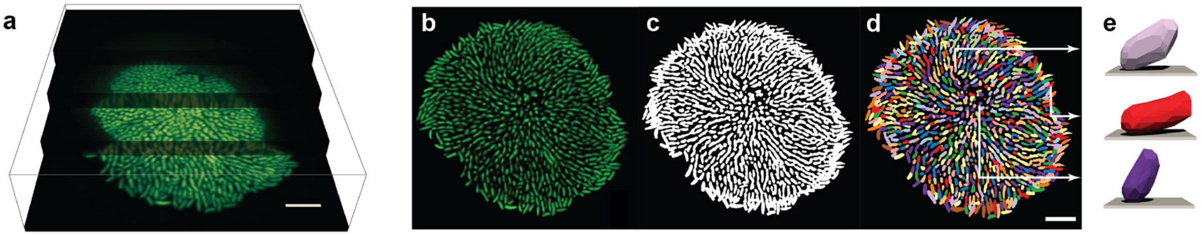

Supplementary Fig. 2| Cell segmentation procedure. a, Representative raw 3D image of a WT* biofilm. **b-d**, Intermediate steps during biofilm segmentation; after deconvolution with a measured point spread function (**b**), after binarization (**c**), after segmentation using an adaptive local thresholding algorithm (**d**). **e**, 3D reconstruction of three different segmented cells at three different locations in the biofilm. Scale bars, 10 μm.

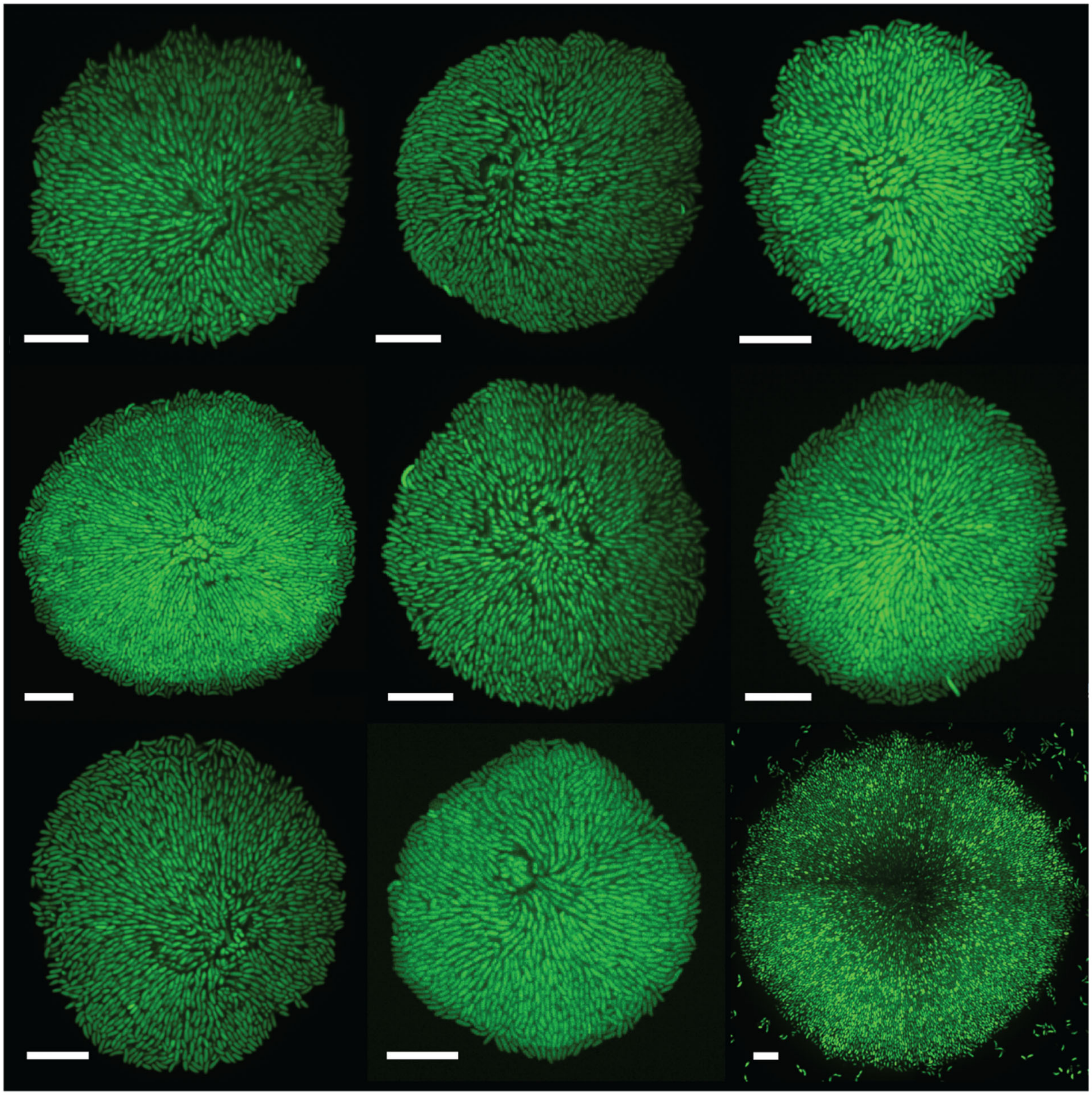

Supplementary Fig. 3| *V. cholerae* biofilms reproducibly form aster patterns. Nine different mature WT* biofilms all exhibiting the aster pattern. The core of the aster, typically defined by the location of the founder cell, does not necessarily coincide with the geometric center of the biofilm due to stochasticity during early biofilm development. Some biofilms also display some local chirality, which could come from noise in cell growth, substrate defects, local configuration variations etc. This is because if a local area grows slower than its surroundings, cells in neighboring areas will grow into this area, resulting in local "bending" of the director field. In the bottom right example, the biofilm is grown for 72 hrs. with fresh growth media replenished at 24 and 48 hrs. Furthermore, the cells are stained using SYTO 9 nulear stain and four adjacent fields of view are stitched together. Scale bars, 10 μm.

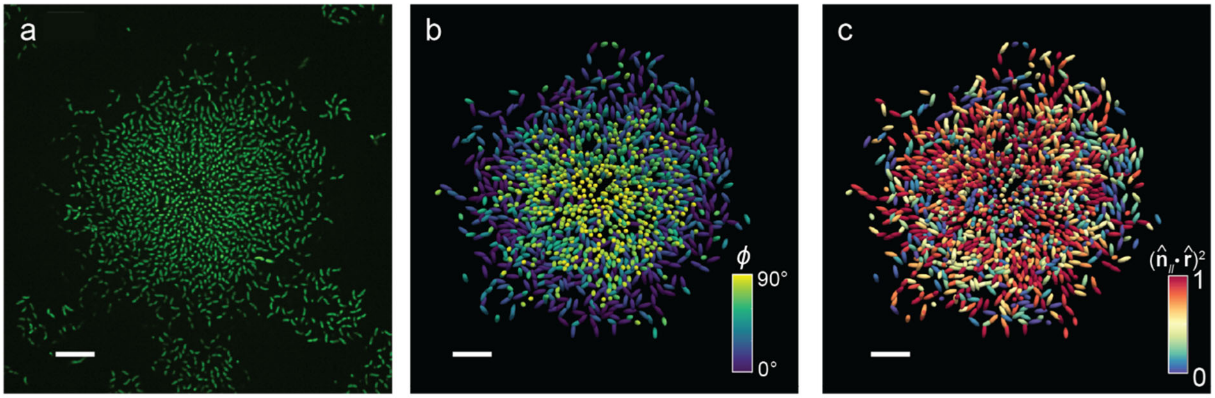

Supplementary Fig. 4| Biofilms in unconfined environments verticalize and develop radial order. a, Cross-sectional view of the basal plane of a WT* biofilm grown without an overlain gel. **b, c**, The same biofilm reconstructed where cells are color-coded by the angle ϕ each cell makes with the substrate (**b**) and the degree of radial alignment $(\hat{\mathbf{n}}_{\parallel} \cdot \hat{\mathbf{r}})^2$ (**c**). We found that cells in unconfined biofilms also tended to align radially ($S = 0.18 \pm 0.05, n = 4$ biofilms), albeit to a lesser extent than confined biofilms ($S = 0.54 \pm 0.07$), which has been reported before[25,26] but never explained. We believe that the same macroscopic ordering mechanism applies generally to biofilms grown in any geometry. In this work, we used a confined geometry to increase the basal area of the biofilm, in order to focus on this emergent organization. Note that cells that do not belong to this cluster are manually removed. Scale bars, 10 µm. Source data are provided as a Source Data file.

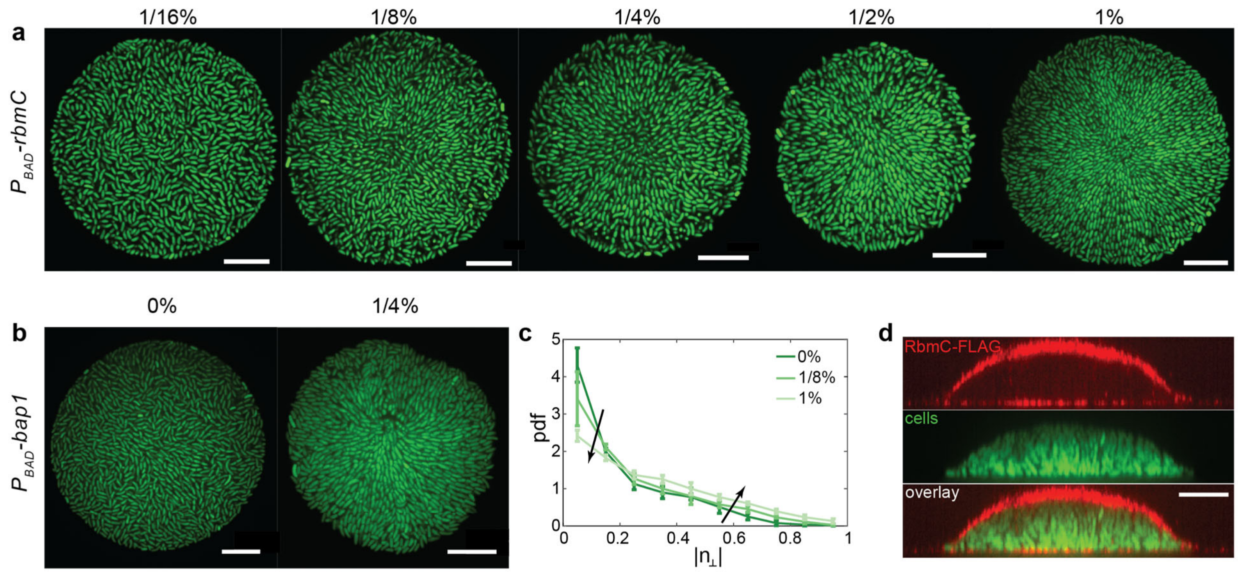

Supplementary Fig. 5| Cell-to-surface adhesion controls macroscopic cell organization. a, Cross-sectional view of the basal layer of biofilms with arabinose-inducible *rbmC* expression. In the presence of different concentrations of arabinose, a transition from disorder-to-order was observed with increasing arabinose. **b**, A similar disorder-to-order transition was seen with arabinose-inducible *bap1* expression. Bap1 and RbmC have been shown to contribute to cell-to-surface adhesion in a partially redundant fashion[25]. **c**, Probability distribution function (pdf) of the degree of verticalization $|n_{\perp}|$ showed a shift to more verticalized cells and less horizontal cells with higher *rbmC* expression controlled by arabinose concentration (error-bars correspond to the mean ± sd; $n = 4, 3, 6$ independent biofilms for 0%, 1/8% and 1% arabinose respectively). **d**, Spatial distribution of RbmC-3×FLAG stained with Cy3-conjugated anti-FLAG antibodies (2 µg/mL; Sigma–Aldrich) shows that RbmC localizes on both the glass and gel surfaces. Scale bars, 10 µm. Source data are provided as a Source Data file.

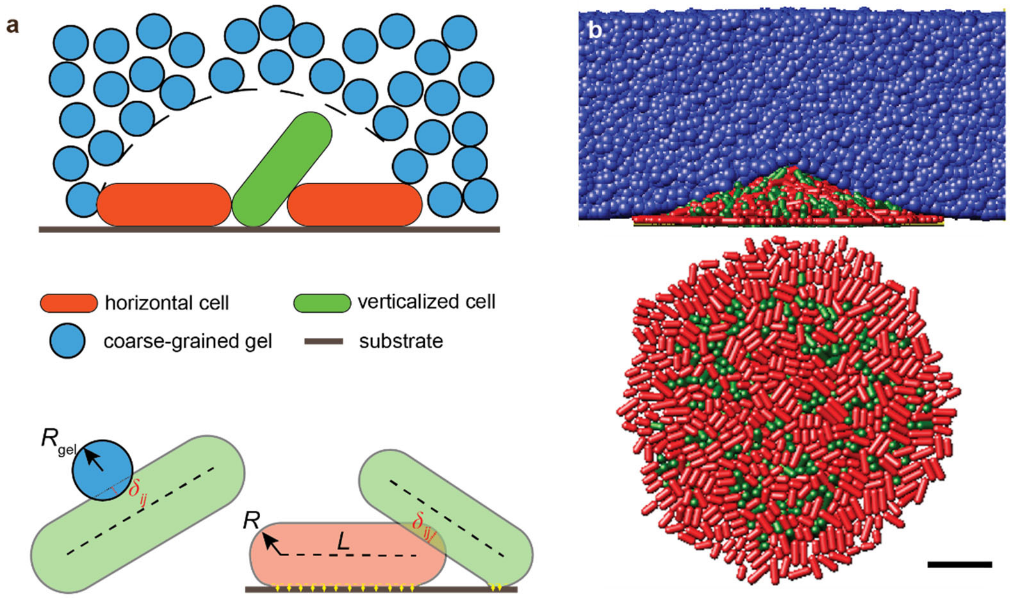

Supplementary Fig. 6| Setup of the agent-based model. a, Schematic illustration of the agent-based simulation consisting of biofilm dwelling cells, which are modeled as growing spherocylinders with length $L(t)$ and radius R, and the surrounding hydrogel, which is modeled using a coarse-grained particle system. **b**, Side view (*Top*) and bottom view (*Bottom*) of a representative simulated biofilm. In the bottom view, gel particles are omitted for clarity. Verticalized cells ($n_{\perp} > 0.5$) are labelled green, and horizontal cells ($n_{\perp} \leq 0.5$) are labelled red. Scale bar, 10 μm.

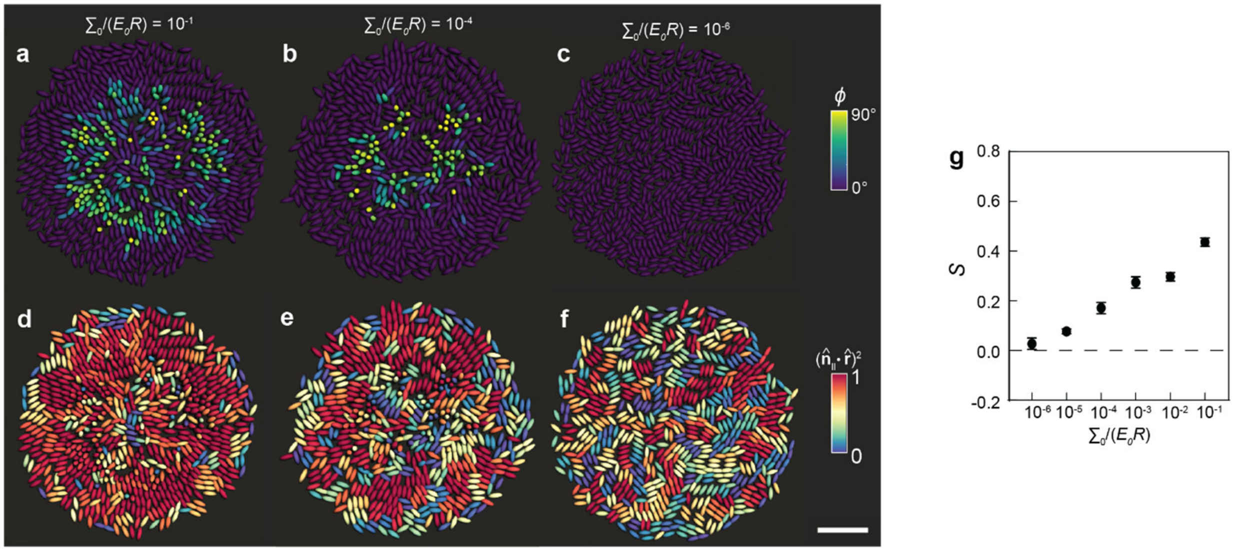

Supplementary Fig. 7| Agent-based models of 3D biofilms with varying adhesion. a-c, Cells in the basal layer, color-coded by the angle ϕ each cell makes with the substrate, in biofilms possessing diffferent adhesion strengths. The dimensionless adhesion strength $\tilde{A} = \Sigma_0/RE_0$ is 10^{-1}, 10^{-4}, and 10^{-6} for **c** to **e**, respectively. **d-f,** The same biofilms as **a-c** with cells color-coded by the degree of radial alignment $(\hat{\mathbf{n}}_{\parallel} \cdot \hat{\mathbf{r}})^2$. Scale bar, 10 µm. **g,** Averaged S in biofilms with different dimensionless adhesion strengths (error-bars correspond to the mean ± sd; $n = 10$ different simulated biofilms). Source data are provided as a Source Data file.

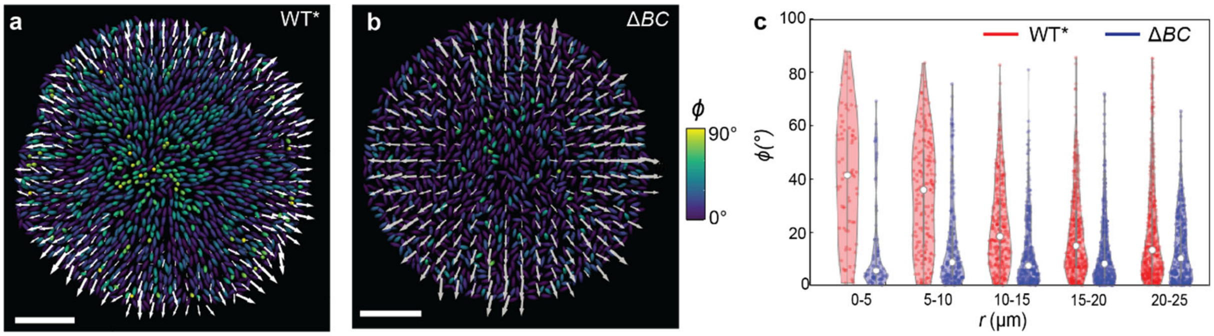

Supplementary Fig. 8| Biofilms formed by nonadherent cells do not support stably verticalized cells in the basal layer. a, b, Reconstructed image of a biofilm from a WT* (**a**) and a Δ*BC* mutant biofilm (**b**), where cells are color-coded by the angle they make with respect to the bottom substrate. Overlain are arrows denoting the measured velocity field. **c**, Violin plot showing the distribution of cell orientations at different radii, for the WT* and Δ*BC* mutant biofilms, respectively. The WT* biofilm was able to sustain verticalized cells leading to a growth void in the middle, whereas the Δ*BC* mutant is unable to sustain verticalized cells, therefore leading to a linear velocity profile. Scale bars, 10 μm. Source data are provided as a Source Data file.

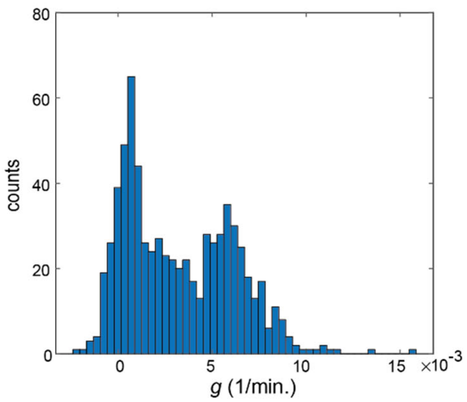

Supplementary Fig. 9| Histogram of apparent in-plane growth rates in a WT* biofilm. Verticalized cells send their offspring into the third dimension and therefore no longer contribute to the expansion of the basal layer. This results in a bimodal distribution of cell growths that are either “growing” or “not growing” in-plane. Source data are provided as a Source Data file.

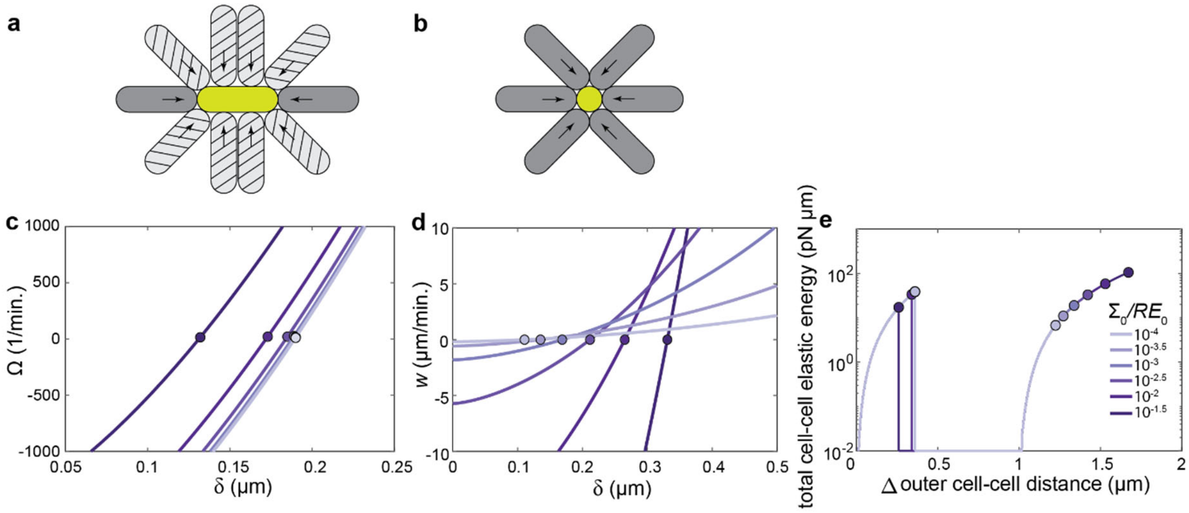

Supplementary Fig. 10| Comparison of energy landscapes for different adhesion energies. a, b, Schematics of the cell configurations for the calculation of the verticalization (**a**) and pinch-off (**b**) instabilities. **c**, **d**, Determination of the instability point for the verticalization (**c**) and pinch-off instabilities (**d**). Regions in which $\Omega > 0, w > 0$ correspond to regions where fixed points are unstable. Circles denote the transition between stable and unstable behavior. Here $\dot{n}_{\perp} = \Omega(\delta)n_{\perp}$ and $\dot{z} = w(\delta, L/2 + 0.999R)$. **e**, Elastic energy due to cell-to-cell contacts in a cell being squeezed incrementally by its neighbors for different dimensionless adhesion energies, $\widetilde{A} = \Sigma_0/RE_0$ (see schematic in Fig. 2f). Circles denote the points at which instabilities occur. Here the energy landscape is calculated using the results of the stability analysis. Note that for $\tilde{A} \leq 10^{-2.5}$ the total elastic energy at verticalization is nearly indistinguishable. Source data are provided as a Source Data file.

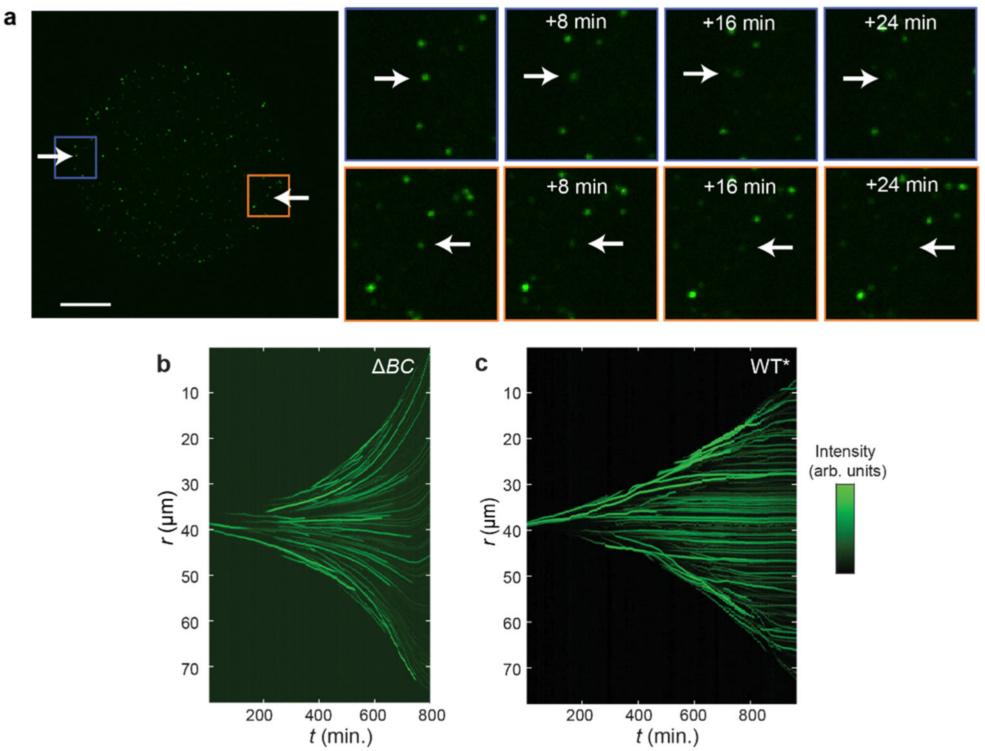

Supplementary Fig. 11| Growth drives cells into the third dimension in the nonadherent mutant. a, Time-lapse imaging of a growing, puncta-labelled Δ*BC* biofilm showing the disappearance of puncta from basal layer over time, due to the corresponding cells being ejected from the basal layer. Scale bar, 10 μm. **b, c** Kymographs of puncta trajectories in a WT* (**b**) and a Δ*BC* mutant biofilm (**c**). In the WT* biofilm a stationary central region develops and expands. In contrast, in the mutant biofilm, no stationary region exists, and some cell trajectories abruptly end due to cell pinch-off.

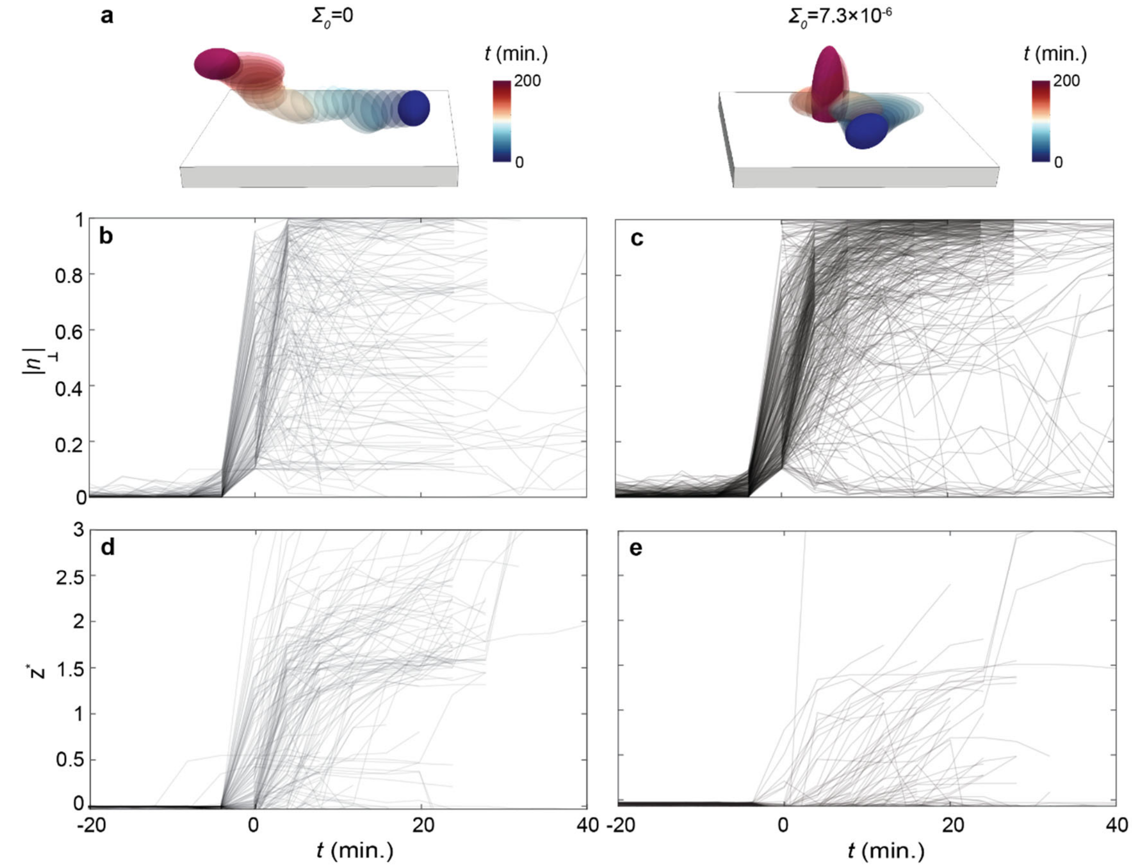

Supplementary Fig. 12| Verticalization and pinch-off dynamics in agent-based simulations. **a**, Example trajectories of a single cell (lineage) from agent-based simulations without adhesion (*Left*) and with adhesion (*Right*). **b**-**e**, Evolution of $n_\perp$ (**b**, **c**) and the cell distance from the surface, z^* (**d**, **e**) around a verticalization event. A verticalization event is defined as the point where $n_\perp$ becomes larger than 0.1 while the cell is still attached to the surface ($z^* = z - R - \frac{L|n_\perp|}{2} \leq 0$). We find that in this particular simulation, after verticalization, adherent cells tend to maintain a vertical configuration and fewer cells leave the surface (11%; 54 out of 495 verticalization events) when compared to nonadherent cells (44%; 144 out of 328 verticalization events). Source data are provided as a Source Data file.

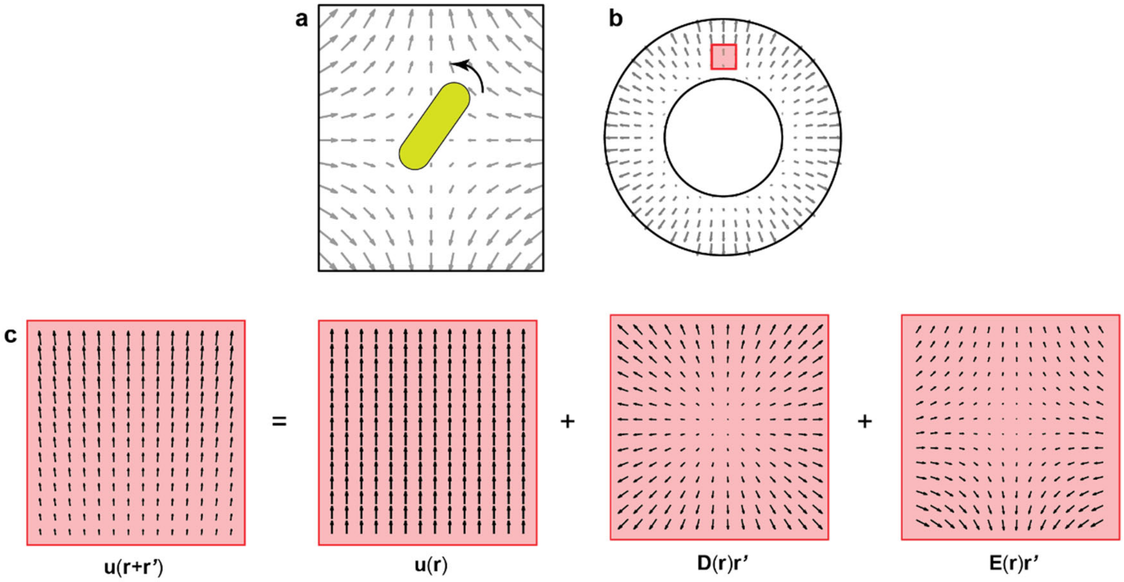

Supplementary Fig. 13| Schematic for the mechanism of flow-induced cell reorientation. a, Schematic showing the response of a rod-shaped bacteria in a straining flow. Under a pure straining flow, bacteria will tend to align along the principal strain axis. **b**, An example flow field assuming a growth void in the center. **c**, Decomposition of the fluid motion into pure translation, expansion, and strain, in the highlighted parcel in **b**. By Taylor expanding, the velocity in the parcel is decomposed in the following way: $\mathbf{u}(\mathbf{r} + \mathbf{r}') = \mathbf{u}(\mathbf{r}) + \mathbf{D}(\mathbf{r})\mathbf{r}' + \mathbf{E}(\mathbf{r})\mathbf{r}'$ where $\mathbf{D} = \frac{1}{2}(\nabla \cdot \mathbf{u})\mathbf{I}$. In the presence of a growth void, each parcel of fluid exhibits straining motion which tends to align cells in the radial direction.

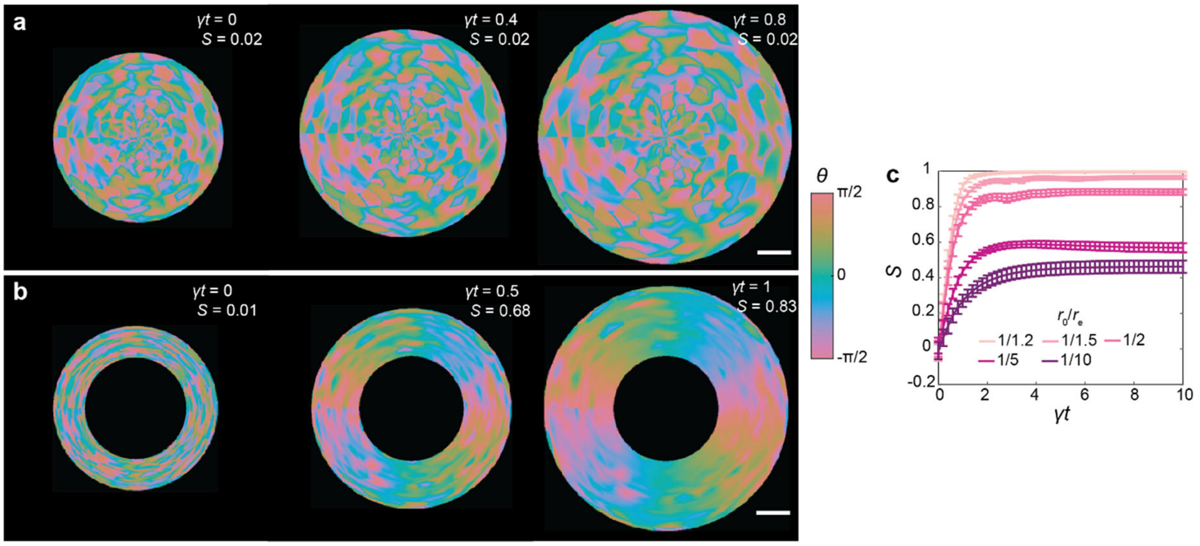

Supplementary Fig. 14| Continuum modelling confirms that a growth void is sufficient to drive radial cell alignment. a, **b**, Evolution of the director field for a biofilm without (**a**) and with (**b**) a growth void. In panel **b** the radius of the void is $r_0 = (3/5)r_\mathrm{e}$, where r_e corresponds to the biofilm size when the growth void is introduced. **c**, Evolution of S averaged over the growing region for different dimensionless initial void sizes r_0/r_e (error-bars correspond to the mean ± sd; $n = 10$ different random initializations). The dimensionless flow alignment parameter $\tilde{L} = \lambda/4q = 1.5$. Radial order emerges over time but plateaus because, unlike the WT* biofilm with an expanding verticalized core, the size of the void is constant in this set of calculations and its effect on cell reorientation is limited to cells near the void due to the $1/r^2$ dependence of the driving force. Scale bars, 10 μm. Source data are provided as a Source Data file.

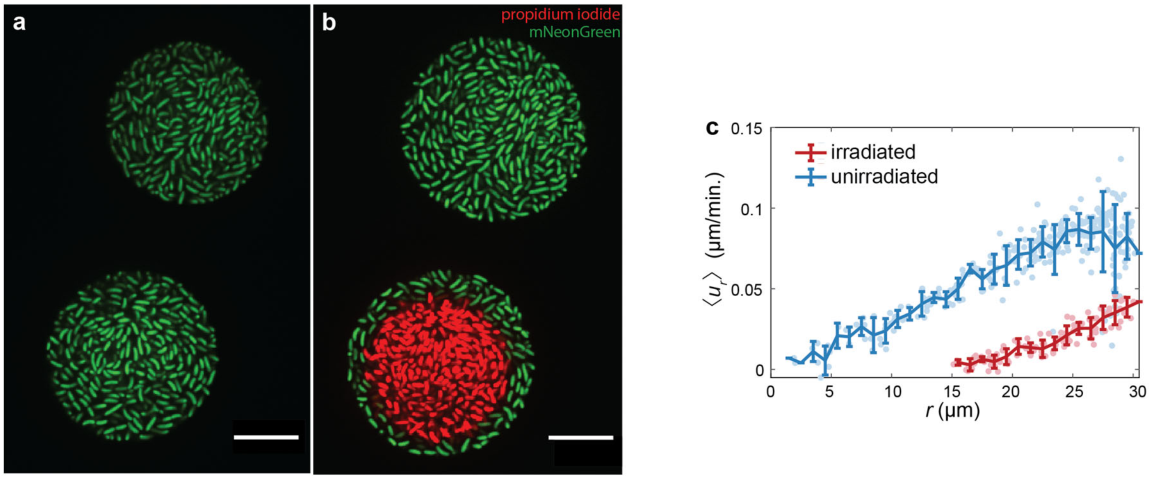

Supplementary Fig. 15| Laser irradiation causes cell death and results in a growth void. a, Two ΔBC biofilms in close proximity before laser irradiation. **b**, The same biofilms after being irradiated in a circular pattern using 405 nm light. Dead cells are stained with propidium iodide. **c**, The measured radial velocity field 1 hour after irradiation (red) and without irradiation (blue) (superimposed error-bars correspond to the mean ± sd of the data binned on 1 μm intervals; $n \geq 2$ cells per bin). Scale bars, 10 μm. Source data are provided as a Source Data file.

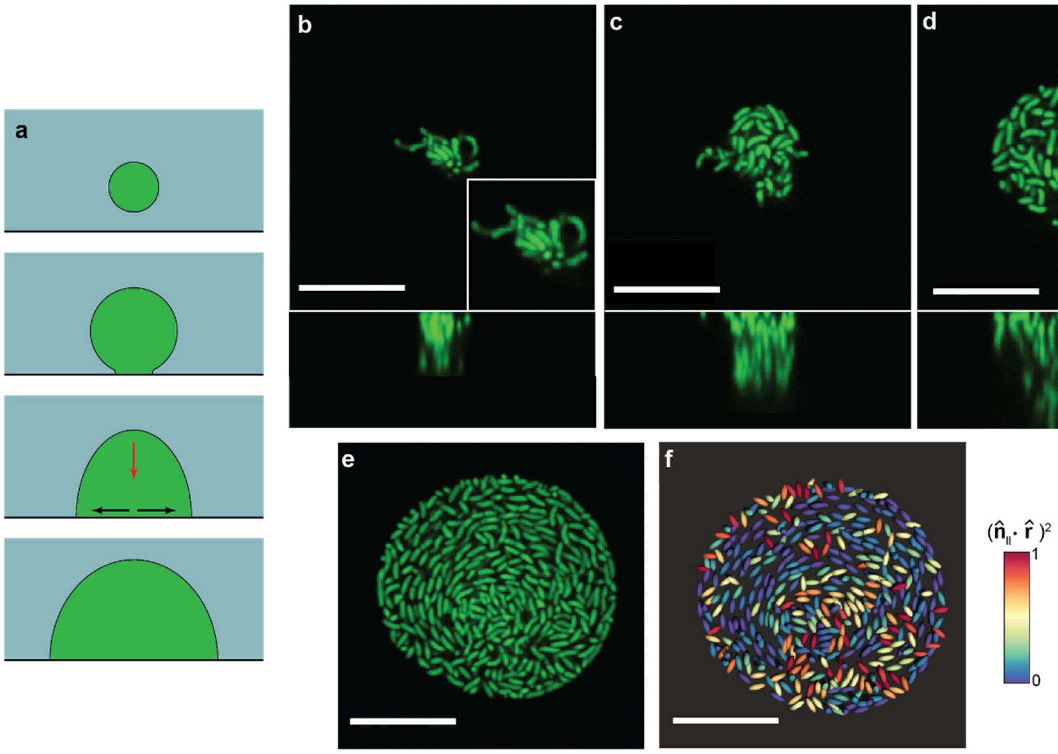

Supplementary Fig. 16| Nonadherent biofilms have the potential to form vortex structures due to non-uniform growth. To probe if vortex structures were possible, we imaged ΔBC biofilms that were initially embedded inside the bulk of the gel instead of starting at the gel-glass interface. This is because, once such a biofilm reached the interface, it preferentially grew at the interface with little increase in biofilm height, leading to excess growth introduced into the basal layer from the bulk, mostly at the center. This process is given schematically in panel **a**. Initially, the biofilm grows completely inside the gel without being in contact with the glass surface. Eventually, the growing biofilm begins to spread between the gel and the glass surfaces and cells grow preferentially along this interface. In this case, excess growth is supplied by cells in the bulk (red arrow) which add to the in-plane growth rate at the center of the basal layer (black arrows). Excess growth at the center gives rise to a negative $f(r,t)$ in Main Text Eq. 2 (see Supplementary Information Section 4 for more discussion), therefore providing a driving force to align cells azimuthally. **b-e**, Time-lapse imaging of a growing ΔBC biofilm in which a vortex-like structure appears ($t = 6, 9, 12, 15$ hrs). A zoom-in image is provided in **b** when the embedded biofilm first touches the glass surface; at this moment cells are randomly oriented. **f**, Reconstructed biofilm from panel **e** where cells are color-coded based on the degree of radial alignment $(\hat{\mathbf{n}}_{\parallel} \cdot \hat{\mathbf{r}})^2$. A value of zero denotes cells that are oriented azimuthally (circumferentially). Source data are provided as a Source Data file.

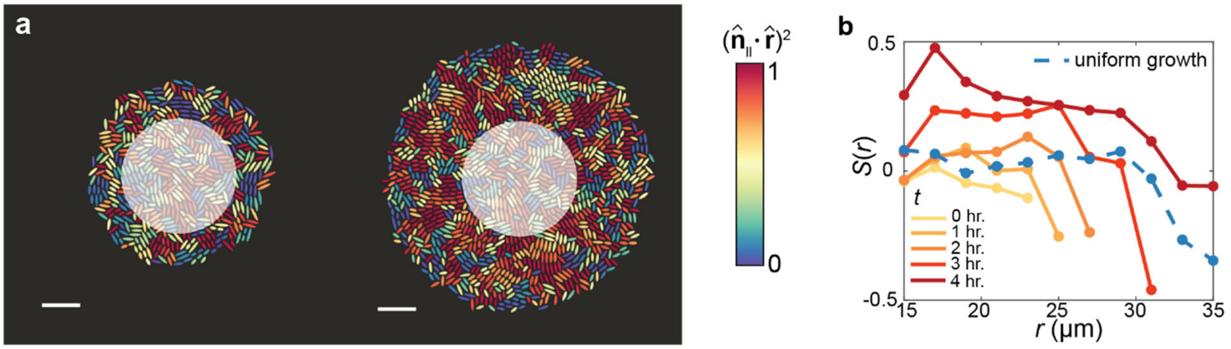

Supplementary Fig. 17| Quasi-2D simulation corresponding to the laser irradiation experiments. a, Configuration of a 2D biofilm (*left*) right before imposing a growth void and (*right*) 4hr after imposing a growth void (highlighted by the white circle). The color denotes the degree to which the cell orientation coincides with the radial director $(\hat{\mathbf{n}}_{\parallel}\cdot\hat{\mathbf{r}})^2$. Scale bars, 10 μm. **b,** Evolution of the azimuthally averaged $S(r)$ in biofilms after the creation of a growth void. Dashed blue line shows the result from a control biofilm without a growth void. Source data are provided as a Source Data file.

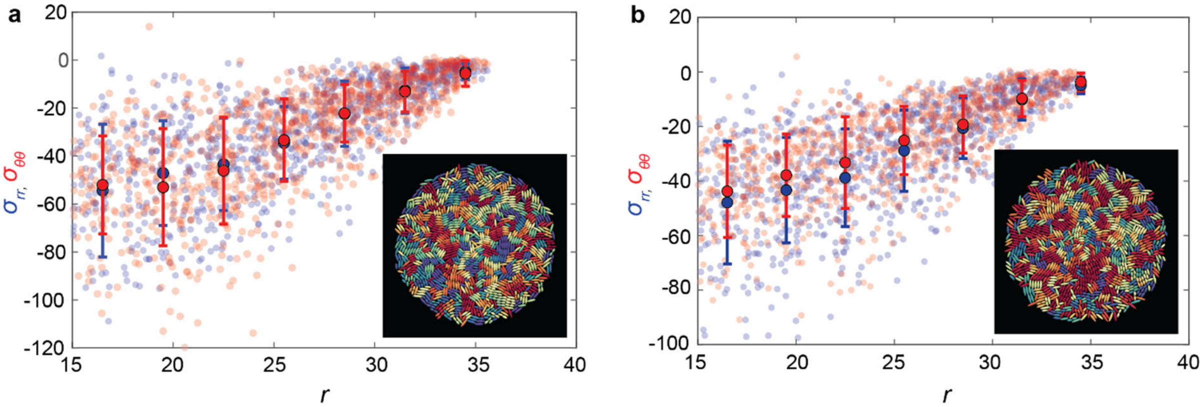

Supplementary Fig. 18| Dependence of stress anisotropy on radial alignment. a-b Measurement of the stress anisotropy in ABS in an isotropic ($S = -0.01$; **a**) and a radially aligned ($S = 0.15$; **b**) 2D biofilm (superimposed error-bars correspond to the mean ± sd of the data binned on 3 μm intervals; $n \geq 20$ cells per bin). In **b** a growth void is introduced to drive radial alignment in the periphery. Inset: The corresponding cell configurations where cells are color-coded by the degree of radial alignment $(\hat{\mathbf{n}}_{\parallel} \cdot \hat{\mathbf{r}})^2$ (red - radially aligned, blue - circumferentially aligned cells). In the radially aligned case, a small systematic amount of anisotropy is observed. Source data are provided as a Source Data file.

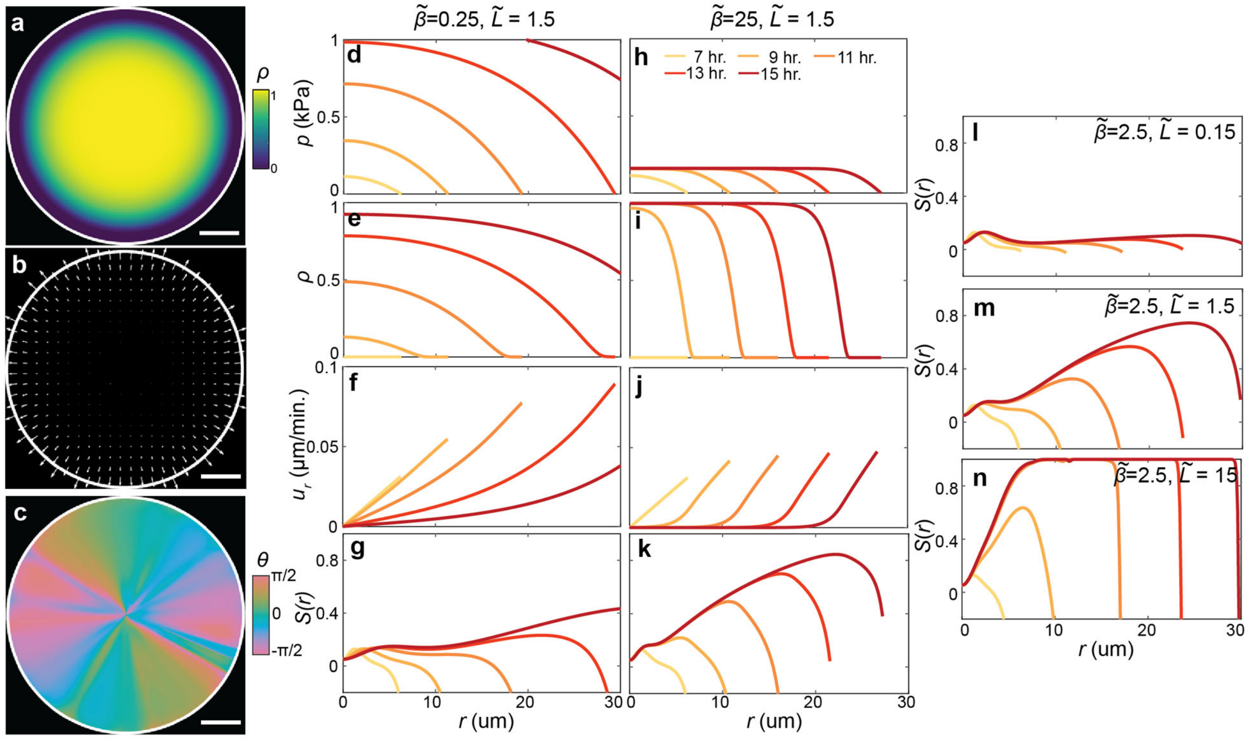

Supplementary Fig. 19| Representative results from two-phase active nematic model. a-c, Representative 2D plots of the fraction of verticalized cells ρ (**a**), the velocity field (**b**), and the director field (**c**) for dimensionless verticalization rate $\tilde{\beta} = 2.5$ and dimensionless flow alignment parameter $\tilde{L} = 1.5$. Scale bars, 10 μm. **d-k**, Results of the model for a small verticalization rate $\tilde{\beta} = 0.25$ (**d-g)** and for a large verticalization rate $\tilde{\beta} = 25$ (**h-k)**. **l-n**, Evolution of S for three different flow alignment rates $\tilde{L}$ while keeping the same $\tilde{\beta} = 2.5$. The colors denote model results at different times in panels **d-n**.

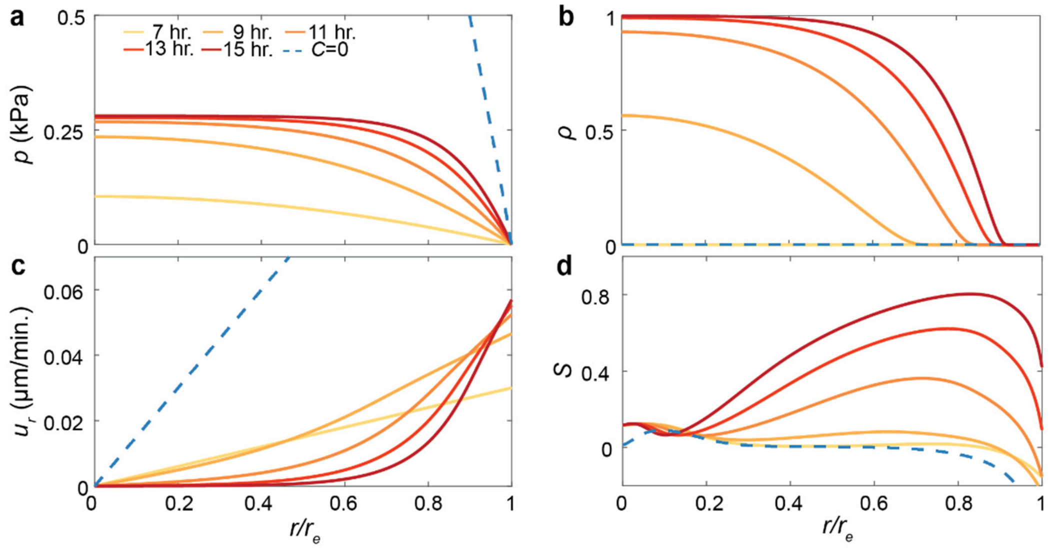

Supplementary Fig. 20| Rescaled two-phase active nematic model. a-d Evolution of pressure p (**a**), fraction of vertical cells ρ (**b**), in plane radial velocity u_r (**c**), and radial order parameter S (**d**). Curves are colored according to time and results for a biofilm that do not verticalize (C = 0) are shown in blue. The data presented here are identical to the data presented in Figure **4a-d** except that the x-axis has been re-scaled by the overall biofilm radius. Source data are provided as a Source Data file.

Table S1: List of the strains used in this study

Strains	Genotype	Source
JN007	*vpvc*^{W240R}, Δ*rbmA*, Δ*vc1807::P*$_{tac}$-*mNeonGreen*-Spec^{R}	This study
JN009	*vpvc*^{W240R}, Δ*rbmA*, Δ*bap1*, Δ*rbmC*, Δ*vc1807::P*$_{tac}$-*mNeonGreen*-Spec^{R}	This study
JN010	*vpvc*^{W240R}, Δ*vpsL*, Δ*vc1807::P*$_{tac}$-*mNeonGreen*-Spec^{R}	This study
JN148	*vpvc*^{W240R}, Δ*rbmA*, Δ*lacZ::P*$_{tac}$-*mNeonGreen-μNS*, Δ*vc1807::P*$_{tac}$-*mScarletI*-Spec^{R}	This study
JN150	*vpvc*^{W240R}, Δ*rbmA*, Δ*bap1*, Δ*rbmC*, Δ*lacZ::P*$_{tac}$-*mNeonGreen-μNS*, Δ*vc1807::P*$_{tac}$-*mScarletI*-Spec^{R}	This study
JN035	*vpvc*^{W240R}, Δ*rbmA*, Δ*bap1*, Δ*rbmC*, Δ*vc1807::P*$_{tac}$-*mNeonGreen*-Spec^{R}, pJY056	This study
JN036	*vpvc*^{W240R}, Δ*rbmA*, Δ*bap1*, Δ*rbmC*, Δ*vc1807::P*$_{tac}$-*mNeonGreen*-Spec^{R}, pJY057	This study
JY489	*vpvc*^{W240R}, Δ*rbmA*, *rbmC-3×FLAG*, Δ*vc1807::P*$_{tac}$-*mNeonGreen*-Spec^{R}	This study
**Plasmid**		
pJY056	Kan^{R}, *araC-P*$_{BAD}$-*rbmC*	[26]
pJY057	Kan^{R}, *araC-P*$_{BAD}$-*bap1*	[26]

Table S2: List of cell parameters used in the agent-based simulations.

$R(\mu\text{m})$	$L_{\text{max}}(\mu\text{m})$	$E_0(\text{Pa})$	$\gamma(\text{s}^{-1})$	$\Sigma_0(\text{Nm}^{-1})$	$\eta_0(\text{Pa}\cdot\text{s})$	$\eta_1(\text{Pa}\cdot\text{s})$
0.8	3.6	300	3.12×10^{-4}	7.3×10^{-6}	20	2×10^{5}

Table S3: List of gel parameters used in the agent-based simulations.

$R_{\mathrm{gel}}(\mu\mathrm{m})$	$E_1(\mathrm{Pa})$	$k_{\mathrm{r}}\ (\mathrm{Nm}^{-1})$	$k_{\zeta}(\mathrm{Nm}^{2})$	$\xi_0(\mu\mathrm{m})$	$\zeta_0(°)$	$\Sigma_1(\mathrm{Nm}^{-1})$
1.0	1500	6×10^{-3}	6.6×10^{-4}	0.6	120	6×10^{-2}

References

1. Beroz, F. *et al.* Verticalization of bacterial biofilms. *Nat. Phys.* **14**, 954–960 (2018).
2. Ostoja-Starzewski, M., Sheng, P. Y. & Alzebdeh, K. Spring network models in elasticity and fracture of composites and polycrystals. *Comput. Mater. Sci.* **7**, 82–93 (1996).
3. Yan, J. *et al.* Mechanical instability and interfacial energy drive biofilm morphogenesis. *eLife* **8**, e43920 (2019).
4. Yan, J. *et al.* Bacterial biofilm material properties enable removal and transfer by capillary peeling. *Adv. Mater.* **30**, 1804153 (2018).
5. Grant, M. A. A., Wacław, B., Allen, R. J. & Cicuta, P. The role of mechanical forces in the planar-to-bulk transition in growing *Escherichia coli* microcolonies. *J. R. Soc. Interface* **11**, 20140400 (2014).
6. You, Z., Pearce, D. J. G., Sengupta, A. & Giomi, L. Mono- to multilayer transition in growing bacterial colonies. *Phys. Rev. Lett.* **123**, 178001 (2019).
7. Su, P.-T. *et al.* Bacterial colony from two-dimensional division to three-dimensional development. *PLoS One* **7**, e48098 (2012).
8. Duvernoy, M.-C. *et al.* Asymmetric adhesion of rod-shaped bacteria controls microcolony morphogenesis. *Nat. Commun.* **9**, 1120 (2018).
9. Cates, M. E. & Tjhung, E. Theories of binary fluid mixtures: from phase-separation kinetics to active emulsions. *J. Fluid Mech.* **836**, P1 (2018).
10. Beris, Antony. N. & Edwards, B. J. *Thermodynamics of flowing systems: with internal microstructure*. (Oxford University Press, 1994).
11. Doostmohammadi, A., Thampi, S. P. & Yeomans, J. M. Defect-mediated morphologies in growing cell colonies. *Phys. Rev. Lett.* **117**, 048102 (2016).

12. Dell'Arciprete, D. *et al.* A growing bacterial colony in two dimensions as an active nematic. *Nat. Commun.* **9**, 4190 (2018).

13. You, Z., Pearce, D. J. G., Sengupta, A. & Giomi, L. Geometry and mechanics of microdomains in growing bacterial colonies. *Phys. Rev. X* **8**, 031065 (2018).

14. Marchetti, M. C. *et al.* Hydrodynamics of soft active matter. *Rev. Mod. Phys.* **85**, 1143–1189 (2013).

15. Copenhagen, K., Alert, R., Wingreen, N. S. & Shaevitz, J. W. Topological defects promote layer formation in *Myxococcus xanthus* colonies. *Nat. Phys.* **17**, 211–215 (2021).

16. Bär, M., Großmann, R., Heidenreich, S. & Peruani, F. Self-propelled rods: insights and perspectives for active matter. *Annu. Rev. Condens. Matter Phys.* **11**, 441–466 (2020).

17. Meacock, O. J., Doostmohammadi, A., Foster, K. R., Yeomans, J. M. & Durham, W. M. Bacteria solve the problem of crowding by moving slowly. *Nat. Phys.* **17**, 205–210 (2021).

18. Volfson, D., Cookson, S., Hasty, J. & Tsimring, L. S. Biomechanical ordering of dense cell populations. *Proc. Natl. Acad. Sci. USA* **105**, 15346–15351 (2008).

19. You, Z., Pearce, D. J. G. & Giomi, L. Confinement-induced self-organization in growing bacterial colonies. *Sci. Adv.* **7**, eabc8685 (2021).

20. Başaran, M., Yaman, Y. I., Yuce, T. C., Vetter, R. & Kocabas, A. Large-scale orientational order in bacterial colonies during inward growth. Preprint at https://arxiv.org/abs/2008.05545 (2021).

21. Cho, H. *et al.* Self-organization in high-density bacterial colonies: efficient crowd control. *PLoS Biol.* **5**, e302 (2007).

22. Sheats, J., Sclavi, B., Cosentino Lagomarsino, M., Cicuta, P. & Dorfman, K. D. Role of growth rate on the orientational alignment of *Escherichia coli* in a slit. *R. Soc. Open Sci.* **4**, (2017).

23. Thijssen, K., Metselaar, L., Yeomans, J. M. & Doostmohammadi, A. Active nematics with anisotropic friction: the decisive role of the flow aligning parameter. *Soft Matter* **16**, 2065–2074 (2020).

24. Liu, S., Shankar, S., Marchetti, M. C. & Wu, Y. Viscoelastic control of spatiotemporal order in bacterial active matter. *Nature* **590**, 80–84 (2021).

25. Berk, V. *et al.* Molecular architecture and assembly principles of *Vibrio cholerae* biofilms. *Science* **337**, 236–239 (2012).

26. Yan, J., Sharo, A. G., Stone, H. A., Wingreen, N. S. & Bassler, B. L. *Vibrio cholerae* biofilm growth program and architecture revealed by single-cell live imaging. *Proc. Natl. Acad. Sci. USA* **113**, e5337-5343 (2016).
