# Supplementary material for: Mechanical forces drive a reorientation cascade leading to biofilm self-patterning
Source: Nat Commun. 2021 Nov 17;12:6632. doi: 10.1038/s41467-021-26869-6 (PMC8599862; doi:10.1038/s41467-021-26869-6)
Supplement: Supplementary file 3 — Description of Additional Supplementary Files [file 41467_2021_26869_MOESM3_ESM.docx]

**Description of Additional Supplementary Files**

**Title: Supplementary Movie 1**

**Description:** A time-lapsed cross-sectional view of the basal plane of a growing WT* biofilm. Imaging began immediately after seeding the founder cell. The total duration of the movie is 20 hr. The scale bar is $10 \mu m$.

**Title: Supplementary Movie 2**

**Description:** Representative results of the agent-based simulations. **(***Left*) Bottom view of a biofilm in which cells are colored red if horizontal ($n_{\perp}\leq0.5$) or green if vertical ($n_{\perp}>0.5$). (*Right*) Side view of the same biofilm (red/green) and surrounding course-grained gel particles (blue). The duration of the movie is 18 hr.

**Title: Supplementary Movie 3**

**Description:** A time-lapsed cross-sectional view of the basal plane of a WT* biofilm with mNeonGreen labelled puncta. Imaging began 8 hr after seeding the founder cell. The total duration of the movie is 16 hr. The scale bar is $10 \mu m$.

**Title: Supplementary Movie 4**

**Description:** Representative results of a quasi-2D agent-based simulation in which a growth void is introduced at the center of a growing biofilm. The movie is slowed down 1.5-fold after the introduction of the growth void. The total duration of the movie is 16 hr.
