# Supplementary material for: Mechanical forces drive a reorientation cascade leading to biofilm self-patterning
Source: Nat Commun. 2021 Nov 17;12:6632. doi: 10.1038/s41467-021-26869-6 (PMC8599862; doi:10.1038/s41467-021-26869-6)
Supplement: Supplementary file 8 — nr-reporting-summary [file 41467_2021_26869_MOESM8_ESM.pdf]

nature research

Corresponding author(s): Jing Yan

Last updated by author(s): Oct 15, 2021

Reporting Summary

Nature Research wishes to improve the reproducibility of the work that we publish. This form provides structure for consistency and transparency in reporting. For further information on Nature Research policies, see our Editorial Policies and the Editorial Policy Checklist.

Statistics

For all statistical analyses, confirm that the following items are present in the figure legend, table legend, main text, or Methods section.

n/a	Confirmed	
☐	☒	The exact sample size (n) for each experimental group/condition, given as a discrete number and unit of measurement
☐	☒	A statement on whether measurements were taken from distinct samples or whether the same sample was measured repeatedly
☐	☒	The statistical test(s) used AND whether they are one- or two-sided<br>*Only common tests should be described solely by name; describe more complex techniques in the Methods section.*
☒	☐	A description of all covariates tested
☐	☒	A description of any assumptions or corrections, such as tests of normality and adjustment for multiple comparisons
☐	☒	A full description of the statistical parameters including central tendency (e.g. means) or other basic estimates (e.g. regression coefficient) AND variation (e.g. standard deviation) or associated estimates of uncertainty (e.g. confidence intervals)
☐	☒	For null hypothesis testing, the test statistic (e.g. F, t, r) with confidence intervals, effect sizes, degrees of freedom and P value noted<br>*Give P values as exact values whenever suitable.*
☒	☐	For Bayesian analysis, information on the choice of priors and Markov chain Monte Carlo settings
☒	☐	For hierarchical and complex designs, identification of the appropriate level for tests and full reporting of outcomes
☒	☐	Estimates of effect sizes (e.g. Cohen's d, Pearson's r), indicating how they were calculated

Our web collection on statistics for biologists contains articles on many of the points above.

Software and code

Policy information about availability of computer code

Data collection	Nikon NIS-Elements AR (version 5.21.02) was used for image acquisition.<br>The agent-based simulations were implemented in the framework of the molecular dynamics simulator LAMMPS (version 22Aug18) available at https://doi.org/10.5281/zenodo.5570887 .
Data analysis	Images were deconvolved using SVI Huygens Essential (version 20.04).<br>Images were analyzed using custom written Matlab codes (version R2018a) available at https://doi.org/10.5281/zenodo.5570867 .<br>Reconstructed biofilm images were rendered using Paraview (version 5.7.0).

For manuscripts utilizing custom algorithms or software that are central to the research but not yet described in published literature, software must be made available to editors and reviewers. We strongly encourage code deposition in a community repository (e.g. GitHub). See the Nature Research guidelines for submitting code & software for further information.

Data

Policy information about availability of data

All manuscripts must include a data availability statement. This statement should provide the following information, where applicable:

- Accession codes, unique identifiers, or web links for publicly available datasets
- A list of figures that have associated raw data
- A description of any restrictions on data availability

Source data for all figures are provided with this manuscript. All other data are available from the corresponding authors upon reasonable request.

Field-specific reporting

Please select the one below that is the best fit for your research. If you are not sure, read the appropriate sections before making your selection.

☒ Life sciences ☐ Behavioural & social sciences ☐ Ecological, evolutionary & environmental sciences

For a reference copy of the document with all sections, see nature.com/documents/nr-reporting-summary-flat.pdf

Life sciences study design

All studies must disclose on these points even when the disclosure is negative.

Sample size	No sample-size calculations were performed. Data were taken from at least three independent biofilm measurements consisting of many thousands of cells or from a single biofilm imaged over time. For those that were imaged over time, repeated measurements were made on independent biofilms to confirm the quantitative behaviour.
Data exclusions	No data were excluded from the analyses.
Replication	Data was generated from repeated experiments. Each experiment was successful and exhibited the same reproducible behavior.
Randomization	Biofilms of comparable size were randomly selected to image.
Blinding	Blinding was not relevant in this study. Identical computational analysis tools were applied to all samples and were agnostic to the exact experimental conditions.

Reporting for specific materials, systems and methods

We require information from authors about some types of materials, experimental systems and methods used in many studies. Here, indicate whether each material, system or method listed is relevant to your study. If you are not sure if a list item applies to your research, read the appropriate section before selecting a response.

Materials & experimental systems

n/a	Involved in the study	
☒	☐	Antibodies
☒	☐	Eukaryotic cell lines
☒	☐	Palaeontology and archaeology
☒	☐	Animals and other organisms
☒	☐	Human research participants
☒	☐	Clinical data
☒	☐	Dual use research of concern

Methods

n/a	Involved in the study	
☒	☐	ChIP-seq
☒	☐	Flow cytometry
☒	☐	MRI-based neuroimaging
